# Elevated SLC7A2 expression is associated with an abnormal neuroinflammatory response and nitrosative stress in Huntington’s disease

**DOI:** 10.1186/s12974-024-03038-2

**Published:** 2024-02-28

**Authors:** Ian D. Gaudet, Hongyuan Xu, Emily Gordon, Gianna A. Cannestro, Michael L. Lu, Jianning Wei

**Affiliations:** Department of Biomedical Science, Charles E. Schmidt College of Medicine, Florida Atlantic University, Boca Raton, FL 33431 USA

**Keywords:** Huntington’s disease, Arginine transporter, SLC7A2, iNOS, NO, Nitrosative stress, Neuroinflammation, Nitrosylation

## Abstract

We previously identified solute carrier family 7 member 2 (SLC7A2) as one of the top upregulated genes when normal Huntingtin was deleted. SLC7A2 has a high affinity for l-arginine. Arginine is implicated in inflammatory responses, and SLC7A2 is an important regulator of innate and adaptive immunity in macrophages. Although neuroinflammation is clearly demonstrated in animal models and patients with Huntington’s disease (HD), the question of whether neuroinflammation actively participates in HD pathogenesis is a topic of ongoing research and debate. Here, we studied the role of SLC7A2 in mediating the neuroinflammatory stress response in HD cells. RNA sequencing (RNA-seq), quantitative RT-PCR and data mining of publicly available RNA-seq datasets of human patients were performed to assess the levels of SLC7A2 mRNA in different HD cellular models and patients. Biochemical studies were then conducted on cell lines and primary mouse astrocytes to investigate arginine metabolism and nitrosative stress in response to neuroinflammation. The CRISPR–Cas9 system was used to knock out SLC7A2 in STHdhQ7 and Q111 cells to investigate its role in mediating the neuroinflammatory response. Live-cell imaging was used to measure mitochondrial dynamics. Finally, exploratory studies were performed using the Enroll-HD periodic human patient dataset to analyze the effect of arginine supplements on HD progression. We found that SLC7A2 is selectively upregulated in HD cellular models and patients. HD cells exhibit an overactive response to neuroinflammatory challenges, as demonstrated by abnormally high iNOS induction and NO production, leading to increased protein nitrosylation. Depleting extracellular Arg or knocking out SLC7A2 blocked iNOS induction and NO production in STHdhQ111 cells. We further examined the functional impact of protein nitrosylation on a well-documented protein target, DRP-1, and found that more mitochondria were fragmented in challenged STHdhQ111 cells. Last, analysis of Enroll-HD datasets suggested that HD patients taking arginine supplements progressed more rapidly than others. Our data suggest a novel pathway that links arginine uptake to nitrosative stress via upregulation of SLC7A2 in the pathogenesis and progression of HD. This further implies that arginine supplements may potentially pose a greater risk to HD patients.

**Supplementary Information:**

The online version contains supplementary material available at 10.1186/s12974-024-03038-2.

## Background

Huntington’s disease (HD) is a fatal monogenetic neurodegenerative disorder with no current cure. Although the genetic mutation of HD, i.e., the abnormal expansion of CAG repeats in the huntingtin gene (*HTT*), was identified almost 30 years ago [1], the molecular pathogenesis of HD remains elusive. HTT is a multifunctional protein, and one important function is to modulate gene transcription [2]. Transcriptional dysregulation is known to be a central pathogenic mechanism in HD [3, 4]. RNA sequencing (RNA-seq) analyses have helped to identify global transcriptomic changes in HD animal models and patients [5]. However, few studies have addressed the functional implications of altered gene expression in HD pathogenesis.

Previously, we knocked out normal HTT in human neuroblastoma SH-SY5Y cells using the CRISPR–Cas9 (clustered regularly interspaced palindromic repeats-CRISPR-associated protein 9) genetic editing approach and identified solute carrier family 7 member 2 (SLC7A2) as one of the most significantly upregulated genes by RNA-seq analysis [6]. SLC7A2 encodes cationic amino acid transporter 2 (CAT2) with a high affinity for l-arginine (Arg), a semi-essential amino acid that plays an important role in various physiological processes, such as cell division, proliferation, wound healing and, most notably, immune functions [7, 8]. Arg is the precursor for the synthesis of nitric oxide (NO), urea, polyamines, proline, glutamate, creatine and agmatine [9]. Among the metabolites of Arg, NO is directly associated with inflammatory responses. Nitric oxide synthase (NOS) catalyzes the conversion of Arg to NO and l-citrulline. There are three isoforms of NOS: neuronal NOS (nNOS), endothelial NOS (eNOS) and inducible NOS (iNOS). Compared to the constitutive expression of nNOS and eNOS, which produce the nanomolar range of NO, iNOS is not normally present in cells but is highly induced upon inflammation. The considerable micromolar range of NO produced by iNOS helps to defend against invading pathogens and is thus critical for the inflammatory response and the innate immune system [10]. Abnormally high NO levels from overproduction of iNOS, on the other hand, can be harmful and are associated with a variety of human diseases with a component of inflammation [10, 11].

Excessive NO, often complicated by simultaneous production of superoxide anions during neuroinflammation, leads to nitrosative stress by generating reactive nitrogen species (RNS) that can post-translationally modify proteins via S-nitrosylation (SNO), the covalent attachment of the NO moiety to the thiol group of free cysteine residues in a protein [12–14]. SNO represents a prominent redox reaction mediating NO signaling under both physiological and pathophysiological conditions [15, 16]. Normally, SNO modulates transcriptional activity, synaptic plasticity, neuronal survival, and ion channel activities [15, 17]. In contrast, aberrant SNO protein expression can initiate pathological signaling cascades that have been implicated in a number of neurodegenerative diseases [18–21] and is a subject of intensive research interest. In HD, nitrosylation of dynamin-related protein-1 (Drp-1), the enzyme responsible for mitochondrial fission, activates its enzymatic activity, leading to increased mitochondrial fragmentation in HD neurons [22].

SLC7A2 is an important regulator of innate and adaptive immunity in macrophages by increasing Arg transport [23, 24]. However, the role of SLC7A2 in brain immune responses remains largely uninvestigated. Based on a single-cell RNA-seq (scRNA-seq) dataset of mouse whole cortex/hippocampus 10× genomics from the Allen Brain Institute (http://portal.brain-map.org/atlases-and-data/rnaseq), SLC7A2/CAT2 is highly expressed in astrocytes and vascular and leptomeningeal cells*.* Since astrocytes are key regulators of neuroinflammation [25, 26], we investigated the role of SLC7A2 in various HD models in response to neuroinflammation in this study. Interestingly, we found that *SLC7A2* transcripts are selectively upregulated in HD cellular models and patients. HD cells exhibit an overactive response to neuroinflammatory challenges, as demonstrated by abnormally high iNOS induction and NO production, leading to increased protein nitrosylation. This process depends on Arg transport and is SLC7A2-dependent since removing extracellular Arg or knocking out SLC7A2 can block the aforementioned increase. Additionally, more mitochondria were fragmented in STHdhQ111 cells in response to neuroinflammation. Last, by analyzing the Enroll-HD periodic patient dataset, we found that HD patients taking Arg supplements progressed more rapidly than others. Taken together, our data suggest a novel pathway that links Arg uptake to nitrosative stress via upregulation of SLC7A2 in the pathogenesis and progression of HD.

## Methods

### Cell culture

Cell lines: STHdhQ7/Q7 (STHdhQ7, CH00097) and STHdhQ111/Q111 (STHdhQ111, CH00095) were originally obtained from Coriell Institute for Medical Research and cultured in Dulbecco’s modified Eagle’s medium (DMEM) supplemented with 10% FBS, 1% glutamine and 1% antibiotic–antimycotic solution at 33 °C in a humidified incubator with 5% CO_2_. All cell lines were used within 20 passages after initial purchase.

Primary mouse astrocytes: Brain cell suspensions were prepared from postnatal day 0–1 WT and HD YAC128 mouse pups. Briefly, pups were quickly decapitated, and whole brains were removed and merged in ice-cold dissection media [10 mM HEPES, pH = 7.4, 1 mM sodium pyruvate, 0.1% glucose in HEPES-buffered saline (HBSS, Invitrogen)]. The meninges were completely removed under a dissection microscope. Brain tissue was then digested with 0.25% trypsin in dissection medium at 37 °C for 20 min. At the end of incubation, digested tissue was further incubated at room temperature for 5 min with 0.04 mg/mL deoxyribonuclease I (DNase I, Sigma Aldrich, USA). After DNase I incubation, the supernatant was removed, and the tissues were triturated with a widebore P1000 pipette tip in complete culture medium (DMEM/F12K medium supplemented with 10% FBS and 1% antibiotic–antimycotic solution). The debris was allowed to settle for 2 min, and then the supernatant containing the dissociated cells was transferred to a new tube. The supernatant was then centrifuged at 200×*g* for 2 min, and the pellet was resuspended in an appropriate volume of complete culture medium for seeding. The medium was changed every 3–4 days, and the cells were maintained at 37 °C with 5% CO_2_. Primary astrocytes were used within 3 passages. All animal procedures were approved by the Institutional Animal Care and Use Committee of Florida Atlantic University and were in compliance with the National Institutes of Health Guidelines for the Care and Use of Laboratory Animals.

### Drug treatment

To induce neuroinflammation, STHdhQ7, Q111 cells and primary mouse astrocytes were incubated with murine interferon-gamma (IFNγ, 100 ng/mL, Peprotech) and/or lipopolysaccharide (LPS, 5 μg/mL, Invitrogen, eBioscience™ LPS solution, 500×) for 24 h. No significant cell death was observed during the treatment. Similar concentrations of LPS and IFNγ were used to induce iNOS expression in brain slice cultures [27]. To block NO production, the iNOS inhibitor 1400W hydrochloride (Cayman Chemical) was added at a final concentration of 10 nM during the IFNγ/LPS incubation.

To deplete extracellular Arg, cells were first washed with phosphate-buffered saline (PBS) once and then incubated with Arg-free medium [DMEM/F12K-based SILAC medium without Arg and lysine (Cat. No. 88370, Invitrogen), supplemented with 0.5 mM l-lysine hydrochloride to match the concentration of l-lysine in the complete medium, 10% FBS and 1% antibiotic–antimycotic solution] overnight in a humidified incubator with 5% CO_2_. The next day, the cells were treated with IFNγ/LPS in Arg-free medium for 24 h as described above. To restore the Arg concentration, 0.699 mM arginine hydrochloride (the same concentration based on the provided DMEM formula) was added during the neuroinflammatory challenge.

### NO production

NO released into the medium was measured in the form of nitrite using the Griess assay according to the manufacturer’s instructions (Cat No. G2930, Promega). Absorbance was measured at 540 nm using a microplate reader (BMG Clario). The nitrite concentration was determined from the standard curve established by the reference nitrite solution. Interpolated negative values were entered as 0 μM.

Intracellular NO production was monitored in real time using a cell-permeable fluorescent NO indicator, DAF-2 DA (4,5-diaminofluorescein diacetate) [28]. Briefly, cells seeded in a black 96-well plate (1 × 10^4^ cells/well) were incubated with 5 μM DAF-2DA and 0.02% F-127 in PBS for 30 min. After incubation, the labeling solution was removed, and the cells were challenged with 100 ng/mL IFNγ and 5 μg/mL LPS in complete culture medium under various conditions as specified. Fluorescence intensity (Ex: 485 ± 20 nm, Em: 532 ± 30 nm) was immediately monitored for 24 h at 15-min intervals at 33 °C in a microplate reader (BMG Clario). For data normalization, the initial fluorescent value (time = 0, *F*_0_) was treated as background and subtracted from each subsequent reading.

### Biotin switch assay

Protein SNO was detected using a well-established biotin switch assay [18, 29] with minor modifications. Briefly, cells with different treatments were lysed in HEN lysis buffer containing 100 mM HEPES (pH 8.0), 1 mM EDTA, 0.1 mM neocuproine, 1% Triton X-100, 1 mM PMSF, 0.15% methyl methanethiosulfonate (MMTS) and Halt™ protease inhibitor cocktail (Cat No. 78430, Thermo Scientific) at 4 °C. After centrifugation to remove undissolved debris, the supernatant was further incubated with 2.5% SDS and 0.15% (v/v) MMTS at 50 °C for 45 min with frequent vortexing to block free cysteine groups. Free MMTS was then removed by acetone precipitation. Protein precipitates were resuspended in HENS buffer (100 mM HEPES, pH = 8.0, 1 mM EDTA and 0.1 mM neocuproine and 1% SDS) and divided into two aliquots. One aliquot was further incubated with 50 mM freshly made sodium ascorbate and 0.25 mg/mL (*N*-[6-(biotinamido)hexyl]-3′-(2′-pyridyldithio)propionamide) (biotin-HPDP) for 4 h with continuous shaking. This is to reduce nitrosylated cysteine to free cysteine using sodium ascorbate so that it can be labeled with biotin-HPDP, a sulfhydryl-reactive biotinylation agent. The other aliquot was incubated with 50 mM NaCl and 0.25 mg/mL biotin-HPDP, serving as the internal control for blocking efficiency. Free biotin-HPDP was removed by acetone precipitation. Protein precipitates were then resuspended in an appropriate volume of HENS buffer. Then, 5× SDS-sample buffer without β-mercaptoethanol was added, and the samples were heated at 95 °C for 5 min.

For Western blotting, approximately 20 μg of sample was separated by SDS-PAGE and transferred to nitrocellulose membranes. For dot blotting, 2 μL of sample was directly spotted on a nitrocellulose membrane. The spotted membrane was further allowed to air-dry for 30 min. Ponceau S was used to stain the total protein. After total protein staining, the membrane was blocked with blocking buffer for fluorescent western blotting (Cat. No. MB-070, Rockland Immunochemicals, Inc.) for 2 h at room temperature and then incubated with IRDye 800CW streptavidin (Cat. No. 926-32230, LI-COR) diluted in Tris-buffered saline with 0.1% Tween 20 (1:5000, TBS-T) for 1 h at room temperature followed by extensive washes in TBS-T. Fluorescent signals were detected with a LI-COR Odyssey Fc system, and the images were quantified with the provided Image Studio software.

### Quantitative RT-PCR

After various treatments, RNA samples were isolated using the Direct-Zol™ RNA miniprep Plus kit (Cat. No. R20711, ZYMO RESEARCH) according to the manufacturer’s instructions. To reduce genomic DNA contamination, a 15-min in-column DNase I treatment at room temperature was performed. Quantitative RT-PCR was performed using the SYBR green method as we previously described [6]. The following primers were used: mouse SLC7A2 (NM_007514)-forward: 5′-TCTATGTTCCCCTTACCCCGA-3′, -reverse: 5′-TGACTGCCTCTTACTCACTCTT-3′; mouse NOS2 (NM_010927)-forward: 5′-GTTCTCAGCCCAACAATACAAGA-3′, -reverse: 5′-GTGGACGGGTCGATGTCAC-3′; and mouse actin (NM_007393)-forward: 5′-GGCTGTATTCCCCTCCATCG-3′, -reverse: 5′-CCAGTTGGTAACAATGCCATGT-3′.

### RNA-seq and bioinformatic analysis

#### RNA-seq

STHdhQ7 and Q111 cells were treated with IFNγ/LPS for 24 h as described above. Nontreated cells were used as controls. After treatment, RNA was isolated using the Direct-Zol™ RNA miniprep Plus kit as described above. RNA samples in triplicate for each group were submitted to LC Sciences (TX, USA) for mRNA sequencing. The poly(A) RNA sequencing library was prepared following Illumina’s TruSeq-stranded-mRNA sample preparation protocol. RNA integrity was checked with an Agilent Technologies 2100 Bioanalyzer. Poly(A) tail-containing mRNAs were purified using oligo-(dT) magnetic beads with two rounds of purification. After purification, poly(A) RNA was fragmented using divalent cation buffer at an elevated temperature. Quality control analysis and quantification of the sequencing library were performed using an Agilent Technologies 2100 Bioanalyzer High Sensitivity DNA Chip. Paired-ended sequencing was performed on Illumina’s NovaSeq 6000 sequencing system.

#### Bioinformatic analysis

First, Cutadapt [30] and in-house Perl scripts (LC Sciences) were used to remove the reads that contained adaptor contamination, low-quality bases and/or undetermined bases. Sequence quality was verified using FastQC (http://www.bioinformatics.babraham.ac.uk/projects/fastqc/). HISAT2 [31] was used to map reads to the mouse genome (GRCm38.r101, ftp://ftp.ensembl.org/pub/release-101/fasta/mus_musculus/dna/). The mapped reads of each sample were assembled using StringTie [32]. Then, all transcriptomes were merged to reconstruct a comprehensive transcriptome using Perl scripts and gffcompare. After the final transcriptome was generated, StringTie [32] and Ballgown (http://www.bioconductor.org/packages/release/bioc/html/ballgown.html) were used to estimate the expression levels of all transcripts. StringTie [32] was used to determine the expression levels of mRNAs by calculating FPKM. mRNA differential expression analysis was performed by the R package DESeq2 [33] between two different groups (and by the R package edgeR [34] between two samples). The mRNAs with a false discovery rate (FDR) below 0.05 and absolute fold change ≥ 2 were considered differentially expressed mRNAs.

### Arginine and its metabolite measurements

STHdhQ7 and Q111 cells were plated in 10-cm culture dishes and treated with IFNγ/LPS as described above. After treatment, the cells were washed with PBS three times and scrape harvested. Cell pellets were snap-frozen and sent to Creative Proteomics (Shirley, NY) for urea cycle analysis by HPLC–MS/MS. Briefly, each cell pellet sample was thawed on ice and subsequently homogenized in 70% acetonitrile on an MM 400 mill mixer at 30 Hz for 3 min with the aid of two metal balls. The samples were then sonicated in an ice water bath for 2 min, followed by centrifugal clarification at 21,000×*g* for 5 min. Fifty microliters of clear supernatant of each sample or each calibration solution was mixed in turn with 50 μL of an isotope-labeled internal standard solution of Arg, ornithine, citrulline and tryptophan, 100 μL of a dansyl solution and 50 μL of a borate buffer. The mixtures were reacted at 40 °C for 30 min. After the reaction, 10 μL aliquots of the resultant solutions were injected into a C18 column (2.1 × 150 mm, 1.8 μm) to run LC-MRM/MS on a Waters Acquity UPLC system coupled to a Sciex QTRAP 6500 Plus mass spectrometer, which was operated in positive-ion mode. The mobile phase for LC separation was (A) 0.1% formic acid in water and (B) 0.1% formic acid in acetonitrile for binary-solvent gradient elution (25% to 80% B over 15 min) at 0.35 mL/min and 55 °C. Concentrations of the analytes were calculated by interpolating the constructed linear-regression calibration curves.

### Knocking out SLC7A2 in STHQ7 and Q111 cells using the CRISPR–cas9 gene editing approach

Knocking out SLC7A2 in STHdhQ7 and Q111 cells was performed using the CRISPR gene knockout kit V2 from Synthego (CA, USA). Three single guide RNA (sgRNA) sequences targeting the mouse *Slc7a2* gene (*Slc7a2*, Gene ID 11988) were designed using the online Synthego tool. The three sgRNAs targeting the second exon of *Slc7a2* were CUUUCGCGCGAUGUCUGAUC, GGUUAAGCAGCGGCAGAGUU and GGGCGCUGGGGUCUACGUCC (Additional file 1: Fig. S1A). Pooled sgRNAs were dissolved in nuclease-free H_2_O at a final concentration of 30 μM. *Streptococcus pyogenes* Cas9 protein with two nuclear localization signals (spCas9 2NLS) was obtained from Synthego and used at a final concentration of 20 μM. sgRNA and spCas9 2NLS at a molar ratio of 3:1 were used to form ribonucleoprotein (RNP) complexes at room temperature according to the manufacturer’s instructions. The RNP complex was then delivered to STHdhQ7 and Q111 cells using the Neon electroporation system (Invitrogen) with the following parameters: 1300 V, 20 ms width, and 2 pulses. Forty-eight hours after electroporation, half of the cells were reseeded for further culturing. The other half was harvested for genomic DNA extraction using QuickExtract™ DNA extraction solution (Epicenter) according to the manufacturer’s instructions. Standard PCR was performed to amplify the SLC7A2 fragment containing the mutations with Q5® high-fidelity DNA polymerase (New England Biolabs). The following primers were used: 5′-TGGGTGCTCTGAACCAAGTA-3′ and 5′-ACAGCTCTCCGACCGTGA-3′ (Additional file 1: Fig. S1A). A single DNA band at 450 bp was confirmed by DNA gel electrophoresis. The PCR product was excised, further column purified (Qiagen) and sent for Sanger sequencing (GENEWIZ). The forward sequencing primer used was 5′-TGCTCTGAACCAAGTATCTATTAAAGAGAG-3′ (Additional file 1: Fig. S1A). The obtained Sanger sequencing files were uploaded to the online CRISPR analysis software Inference of CRISPR Edits (ICE) from Synthego (http://ice.synthego.com) to determine the KO efficiency and type of edits (Additional file 1: Fig. S1B). Cells were further diluted to a single-cell suspension and cultured in 96-well plates to confluency. Genomic DNA from a fraction of cells in each well was extracted and used for PCR analysis to amplify the SLC7A2 fragment containing the mutations as described above. Clones with large fragment deletions were further expanded in culture, and the mutation was characterized by Sanger sequencing. Three independent clones with a large *Slc7a2* fragment deletion were used for further experiments in this study.

### Mitochondria dynamics and image analysis

FLX1.8-CMV-MitoTurboRFP plasmids (Addgene, plasmid #28131) were transfected into STHdhQ7 and Q111 cells using the standard Lipofectamine 2000 transfection method to label mitochondria. To perform live-cell imaging, transfected cells were directly seeded in a μ-Slide 8-well chamber slide (Ibidi, Cat #80806). Twenty-four hours after seeding, 100 ng/mL IFNγ and 5 μg/mL LPS were added, and the cells were further incubated for 24 h. Nontreated cells were used as controls. Mitochondrial dynamics were recorded by live-cell imaging using a laser scanning confocal microscope (Nikon A1R) equipped with a Tokai Hit Stage top incubator. Time-lapse images on transfected cells with healthy morphologies were taken with a 60× oil objective (CFI Plan Apochromat Lambda 60× Oil, numerical aperture = 1.4) at a rate of 1 frame per second for 2 min. The acquired frame size was 1024 × 1024 pixels with 16-bit depth and downscaled to 512 × 512 pixels with 8-bit depth using Fiji for further image analysis. Mitochondrial morphology and movements were then analyzed using a Mitometer as described [35]. The major axis length of each mitochondrial track was used as the mitochondrial length. An average of 200 individual mitochondrial tracks were analyzed from each cell. The median length of all mitochondrial tracks from a single cell was calculated and used as the median mitochondrial length of that cell. The ratio of fission and fusion events was calculated. Between 4–6 cells were analyzed for each condition.

### Western blot

Approximately 20 μg of sample lysates were separated by SDS-PAGE and transferred to nitrocellulose membranes. The following primary antibodies were used: iNOS, mouse monoclonal anti-actin (1:1000, Cat. No. sc-47778, Santa Cruz Biotechnology), and rabbit polyclonal GAPDH (1:1000, Cat. No. 10494-1-AP, Proteintech). The secondary antibodies used were goat anti-rabbit Alexa Fluor Plus 800 (Cat. No. A32735, 1:8000, Invitrogen) and goat anti-mouse Alexa Fluor 680 (Cat. No. A21058, 1:5000, Invitrogen). Fluorescent signals were detected with a LI-COR Odyssey Fc system, and the images were quantified with the provided Image Studio software.

### Enroll-HD human patient dataset analysis

Data used in this work were generously provided by the participants in the Enroll-HD study and made available by CHDI Foundation, Inc. Enroll-HD is a global clinical research platform designed to facilitate clinical research in Huntington’s disease. Core datasets are collected annually from all research participants as part of this multi-center longitudinal observational study. Data are monitored for quality and accuracy using a risk-based monitoring approach. All sites are required to obtain and maintain local ethical approval. Enroll-HD version PDS5 (periodic dataset 5) was used for this study, which originated from the Enroll-HD electronic data capture database on October 31, 2020, at 23:00 UTC. The tidyverse package in R was used to extract relevant information from the database. Specifically, the terms “arginine” and “cysteine” were used to filter HD patients taking arginine or cysteine supplements, respectively. Patients who did not take arginine or cysteine were labeled as “Others”. We further examined the filtered data and confirmed that no patients took both arginine and cysteine.

To appraise the effect of Arg or Cys supplement on disease progression, propensity score matching to assess sample balance and decide matching groups for further statistical analysis was performed using the MatchIt package [36] in R. It was used to analyze the Enroll-HD datasets to account for confounding factors [37]. Specifically, we performed the nearest neighbor propensity score matching without replacement with a propensity score estimated using logistic regression of the treatment on the covariates, including age, CAG repeats, sex, and race.

To estimate the supplement effect on disease progression and its standard error, we fit a linear model with motor score, TFC score or MMSE total score as the outcome and the supplement, covariates and their interaction as predictors and included the full matching weights in the estimation. The lm() function was used to fit the outcome, and the comparison() function in the marginaleffects package was used to perform g-computation in the matched sample to estimate the average treatment effect in the supplement group. A cluster-robust variance was used to estimate its standard error (SE) with matching stratum membership as the clustering variable.

The motor score, total functional capacity (TFC) score and Mini-Mental State Exam (MMSE) total score recorded from a single patient at each visit were then plotted. The regression line was calculated by applying linear regression using the geom_smooth() function with the ggplot package in R.

### Statistical analysis

All data are expressed as the mean ± S.E.M. and were subjected to unpaired *t* tests or one-way or two-way ANOVA followed by the post hoc multiple comparison test using the GraphPad Prism software statistical package 10.0 as specified. The criterion for significance was set at *p* ≤ 0.05.

## Results

### SLC7A2 expression is upregulated in HD at the transcriptional level

**Fig. 1:**
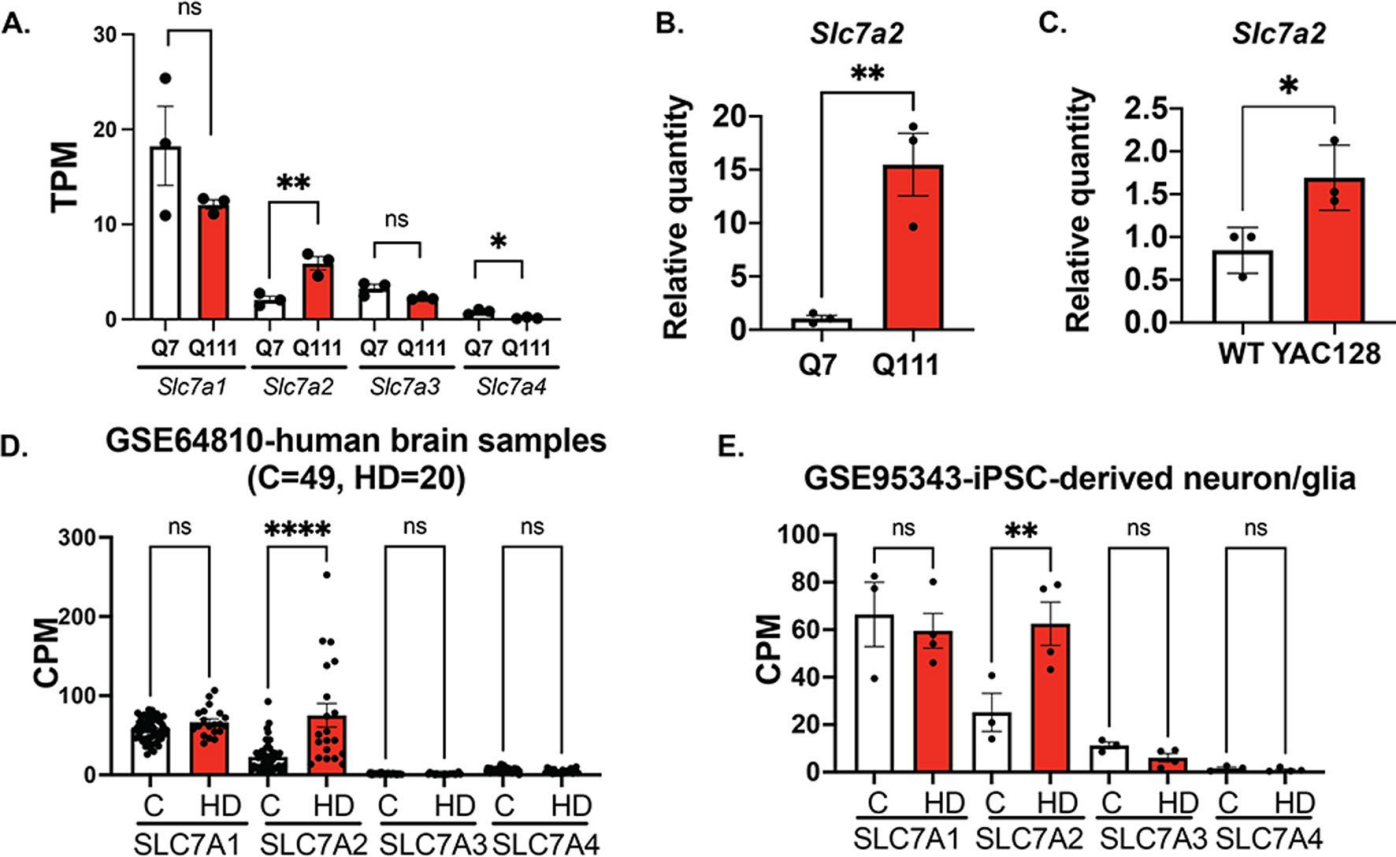
The SLC7A2 mRNA level is upregulated in HD. **A** Quantification of *Slc7a1*-*a4* transcripts in transcripts per million (TPM) in STHdhQ7 and Q111 cells by RNA-seq analysis. **p = 0.0082, *p = 0.015. **B**, **C** qRT-PCR analysis of *Slc7a2* mRNA levels in STHdhQ7 and Q111 cells (**B**, **p = 0.0081) and primary WT and HD astrocytes (**C**, *p = 0.034). In **A**–**C**, unpaired *t* test, ns, not significant. **D** Quantification of *SLC7A1-A4* transcripts in counts per million (CPM) in healthy individuals and HD patients by data mining from GSE64810. ****p < 0.0001. **E** Quantification of *SLC7A1-A4* transcripts in CPM in healthy individuals and HD iPSC-derived neuron/glia culture by data mining from GSE95343. **p = 0.0038, *ns*, not significant. In **D**, **E**, one-way ANOVA followed by Šidák’s multiple comparisons analysis. *ns* not significant

We recently reported that *SLC7A2* transcripts were significantly upregulated (> 15-fold increase) in human neuroblastoma SH-SY5Y cells when normal HTT was knocked out using the CRISPR–Cas9 system [6], suggesting that normal HTT may functionally repress *SLC7A2* transcription and that this function could be potentially affected by muHTT. We therefore asked whether *SLC7A2* mRNA expression is altered in HD in this study. We first performed RNA-seq analysis on STHdhQ7 and STHdhQ111 cells, the two commonly used immortal mouse striatal HD cell lines. Only *Slc7a2* transcripts were upregulated by twofold in STHdhQ111 cells compared to STHdhQ7 cells, but not other family member proteins, *Slc7a1, a2* and *a4* (Fig. 1A)*.* This is consistent with two other independent RNA-seq studies of STHdhQ7 and Q111 cells, GSE84055 [38] and GSE146673 [39, 40]. We further validated the increase in *Slc7a2* mRNA expression in STHdhQ111 cells by quantitative RT‒PCR (qRT‒PCR, Fig. 1B). Based on the scRNA-seq dataset of mouse whole cortex/hippocampus 10× genomics from the Allen Brain Institute, *Slc7a2* is highly expressed in astrocytes. This prompted us to further analyze *Slc7a2* mRNA expression in primary astrocytes prepared from WT and YAC128 pups. Similarly, *Slc7a2* mRNA was significantly increased in YAC128 astrocytes compared to WT astrocytes (Fig. 1C). To extend our observation to HD patients, we performed data mining of existing Gene Expression Omnibus (GEO) datasets for HD patients. Analysis of multiple independent RNA-seq datasets of human brain samples revealed that *SLC7A2 transcripts* were selectively elevated in HD patients, including GSE64810 (grade 3/4 prefrontal cortex Brodmann area 9 (BA9) [41] shown in Fig. 1D), GSE129473 (symptomatic BA9 [42]) and Al-Dalahmah et al. (single-cell RNA-seq of astrocytes from grade 3/4 cingular cortex [43]). By examining the scRNA-seq data from postmortem caudate putamen tissue of grade 2–4 HD and matched unaffected controls (GSE152058) [44], we further noticed that there is a moderate but significant increase in *SLC7A2* mRNA in astrocytes (log2FC = 0.24, FDR = 9 × 10^–66^). Moreover, a significant increase in *SLC7A2 mRNA* expression in HD-induced pluripotent stem cell (iPSC)-derived neuron/glia mixed culture (GSE95343) was also noted [45] (Fig. 1E). In both Fig. 1D, E, counts per million (CPM) were recalculated from the specified datasets using GREIN, an interactive web platform for reanalyzing GEO RNA-seq data [46]. Taken together, these data strongly indicate that *SLC7A2* mRNA is selectively elevated in various HD models, including immortal striatal cell lines, animal models, HD patients and iPSC-derived models. Due to the lack of a reliable antibody to detect SLC7A2 by Western blot, we were unable to test whether SLC7A2 is increased at the protein level. Since it has been reported that SLC7A2 is an important regulator of innate and adaptive immunity [23, 24], we performed functional studies to investigate whether the selective increase in SLC7A2 in HD affects the neuroinflammatory responses in HD.

### Extracellular Arg enhances iNOS and NO production in HD cells upon neuroinflammatory insult

**Fig. 2:**
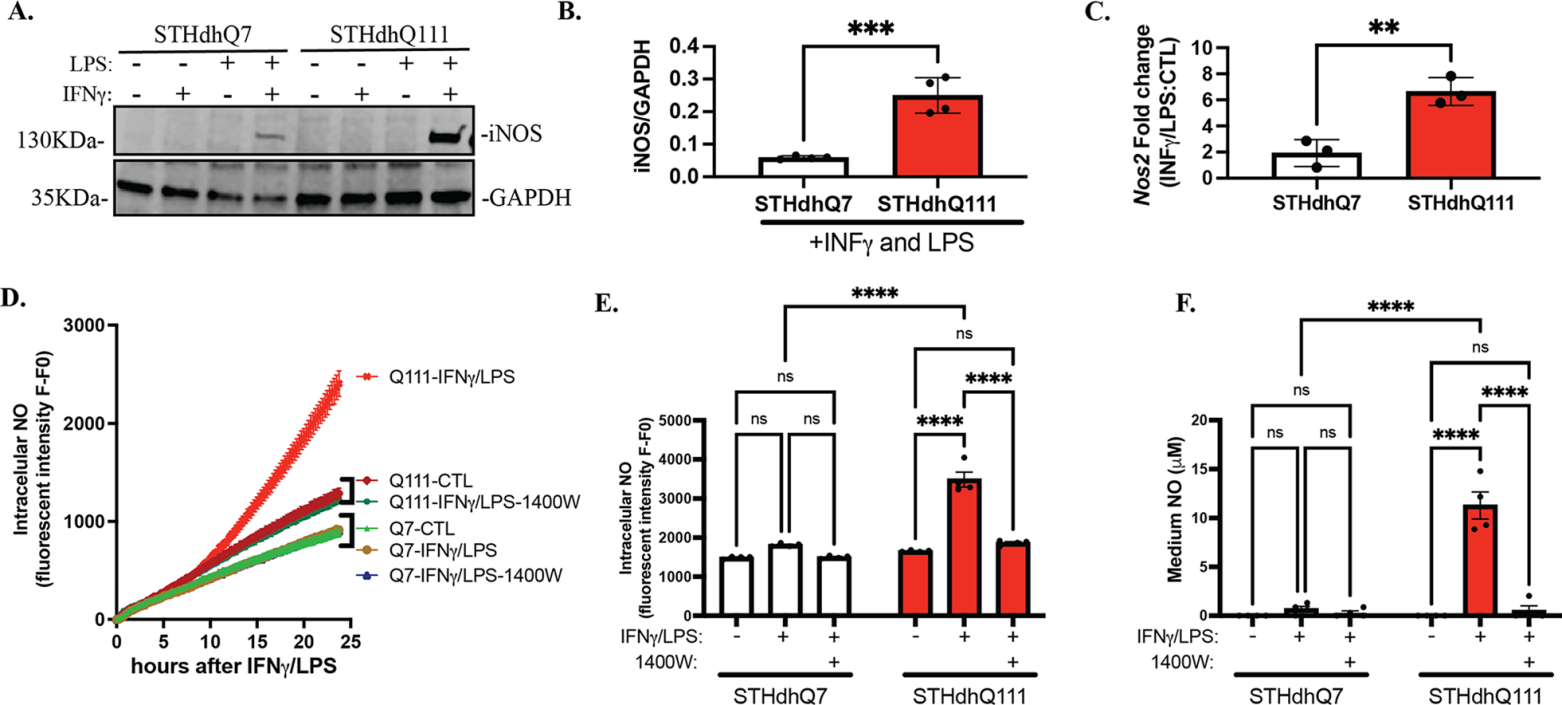
STHdhQ111 cells demonstrate abnormally high iNOS and NO production in response to neuroinflammatory insults. Cells were treated with 100 ng/mL IFNγ and/or 5 μg/mL LPS for 24 h. Nontreated cells were used as controls. **A** Western blot analysis of iNOS expression in STHdhQ7 and Q111 cells under various conditions as indicated. GAPDH was used as the loading control. **B** Quantification of iNOS expression in the presence of IFNγ/LPS treatment from four independent western blots by densitometry analysis. ***p = 0.0005. **C** Quantification of changes in iNOS (*Nos2*) mRNA levels in STHdhQ7 and Q111 cells after 24 h of IFNγ/LPS treatment by qRT-PCR analysis. **p = 0.0052. In **B**, **C**, unpaired *t* test. **D** Intracellular NO production in real time monitored by DAF-2 DA in STHdhQ7 and Q111 cells under various conditions as indicated. *N* = 3 per condition. **E** Quantification of intracellular NO levels 24 h post-challenge in STHdhQ7 and Q111 cells under various conditions as indicated. ****p < 0.0001. **F** Quantification of medium NO levels 24 h post-challenge in STHdhQ7 and Q111 cells under different conditions as indicated. ****p < 0.0001. In **E**, **F**, two-way ANOVA followed by Tukey’s multiple comparisons test. *ns* not significant

To establish an effective neuroinflammatory challenge in STHdhQ7 and Q111 cells, we treated cells with 100 ng/mL IFNγ and/or 5 μg/mL LPS for 24 h. iNOS induction, a commonly used indicator of neuroinflammation, was assessed by Western blotting. Only the combination of IFNγ and LPS induced iNOS expression in both cell types (Fig. 2A). In sharp contrast to STHdhQ7 cells, STHdhQ111 cells demonstrated an over twofold increase in iNOS levels (Fig. 2A, B). This increase was mainly due to the transcriptional upregulation of iNOS (*Nos2*) mRNA, as revealed by qRT-PCR analysis (Fig. 2C). Since iNOS catalyzes the conversion of Arg to NO, we next measured NO production. Intracellular NO levels were monitored using DAF-2 DA, a cell-permeable fluorescent NO indicator [28], and released NO in the medium in the form of nitrite was measured by Griess assay. The level of intracellular NO started to rise only in STHdhQ111 cells ~ 10 h after INFγ/LPS treatment (Fig. 2D). At 24 h post-challenge, intracellular NO levels were significantly higher in STHdhQ111 cells but not in STHdhQ7 cells (Fig. 2E). In parallel, medium NO levels were also significantly elevated in challenged STHdhQ111 cells (Fig. 2F). In both assays, increased NO production in challenged STHdhQ111 cells was blocked by 1400W hydrochloride (Fig. 2D–F), a specific inhibitor of iNOS, indicating that this process is iNOS-dependent.

**Fig. 3:**
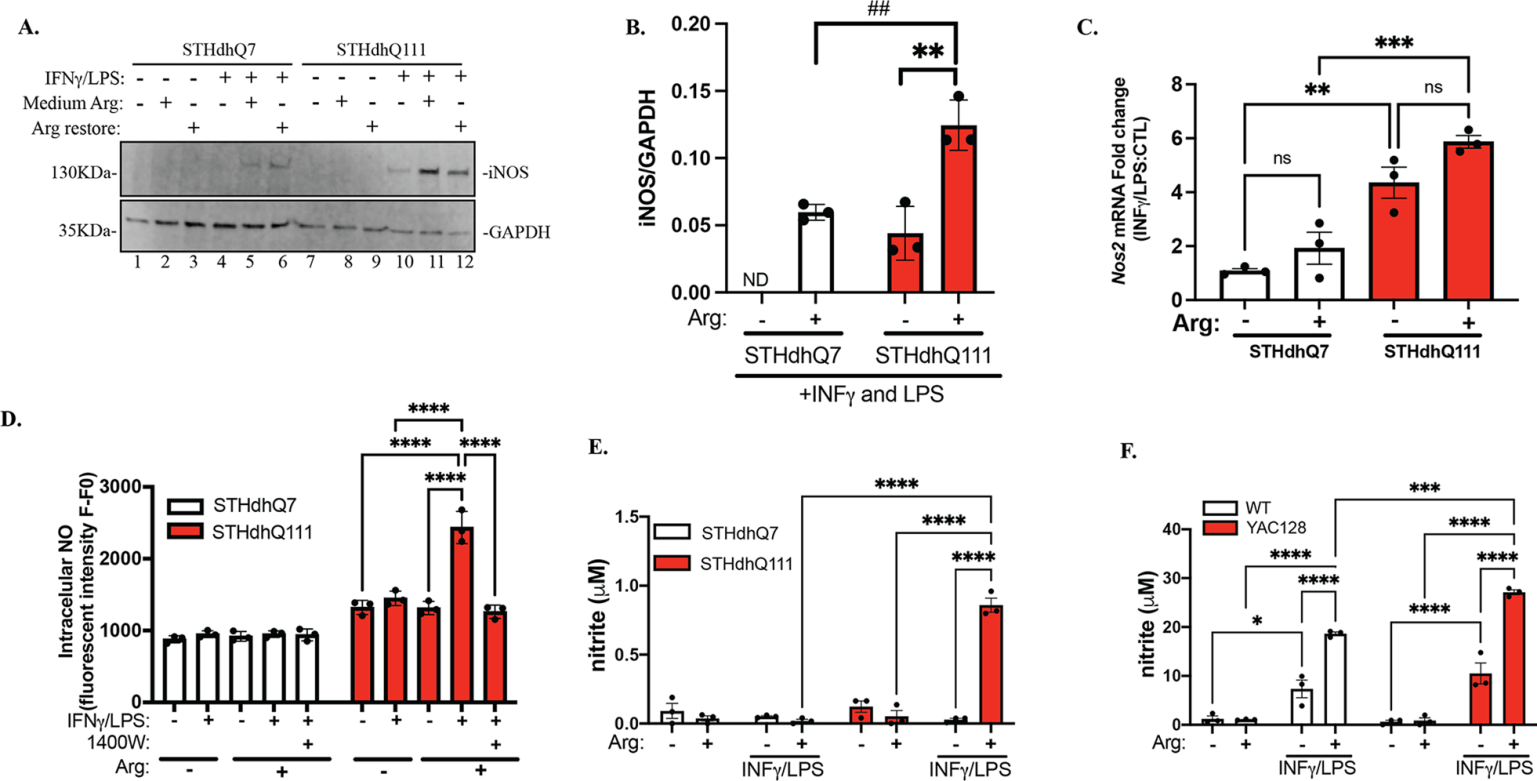
Extracellular Arg regulates IFNγ/LPS-mediated iNOS induction and NO production in HD cells. Cells were first incubated in Arg-free or complete medium overnight. The next day, the cells were treated with IFNγ/LPS for 24 h. To restore Arg under Arg-free conditions, 0.699 mM Arg hydrochloride was added during IFNγ/LPS treatment. **A** Western blot analysis of iNOS expression in STHdhQ7 and Q111 cells under various conditions as indicated. GAPDH was used as a loading control. **B** Quantification of iNOS expression in the presence or absence of extracellular Arg during IFNγ/LPS treatment from three independent Western blots by densitometry analysis. **p = 0.0047, ^##^*p* = 0.0071, *ND* not determined. Unpaired *t* test. **C** Quantification of changes in *Nos2* mRNA expression in the presence or absence of extracellular Arg during IFNγ/LPS treatment by qRT-PCR analysis. **p = 0.031, ***p = 0.0009. **D** Quantification of intracellular NO levels 24 h after IFNγ/LPS treatment under different conditions as indicated in STHdhQ7 and Q111 cells. ****p < 0.0001. **E** Quantification of medium NO levels 24 h after IFNγ/LPS treatment under various conditions as indicated in STHdhQ7 and Q111 cells. ****p < 0.0001. **F** Quantification of medium NO levels 24 h after IFNγ/LPS treatment under various conditions as indicated in WT and HD mouse primary astrocytes. *p = 0.0151, ***p = 0.0007, ****p ≤ 0.0001. In **C**–**F**, two-way ANOVA followed by Tukey’s multiple comparisons test. *ns* not significant

It is reported that extracellular Arg translationally regulates iNOS expression in primary astrocytes [47]. We wondered whether extracellular Arg levels regulate iNOS induction and NO production in our HD models. Strikingly, in the presence of INFγ/LPS, depletion of Arg from the medium significantly reduced iNOS expression in both cell types (Fig. 3A, lane 4 and lane 10 for STHdhQ7 and Q111, respectively) compared to cells cultured in normal complete medium (Fig. 3A, lane 5 and 11 for Q7 and Q111, respectively) or cells that received Arg during INFγ/LPS challenge (Fig. 3A, lane 6 and 12 for STHdhQ7 and Q111, respectively). Interestingly, despite the remarkable reduction in iNOS protein expression, *Nos2* mRNA was still upregulated in INFγ/LPS-treated cells with Arg-free medium (Fig. 3C). Therefore, extracellular Arg levels seem to regulate the expression of iNOS at the translational level. Restoration of extracellular Arg levels during INFγ/LPS treatment increased iNOS translation in both cell types but to a significantly higher extent in STHdhQ111 cells (Fig. 3B). As expected, depletion of extracellular Arg abolished the increase in intracellular NO (Fig. 3D) and medium NO (Fig. 3E) production in response to INFγ/LPS treatment in STHdhQ111 cells, which could be restored by adding Arg back to the medium during the challenge. This is iNOS-dependent since it was blocked by 1400W hydrochloride (Fig. 3D). Finally, we measured medium NO production in HD primary mouse astrocytes in the presence or absence of extracellular Arg, and similar results were obtained (Fig. 3F). Taken together, these data strongly suggest that extracellular Arg translationally regulates iNOS expression and subsequently increases NO production in HD cells in response to neuroinflammatory challenge, suggesting that the continuous transport of Arg across the plasma membrane into the cells is essential for this process.

### Knocking out SLC7A2 attenuates iNOS and NO production in HD cells after INFγ/LPS treatment

**Fig. 4:**
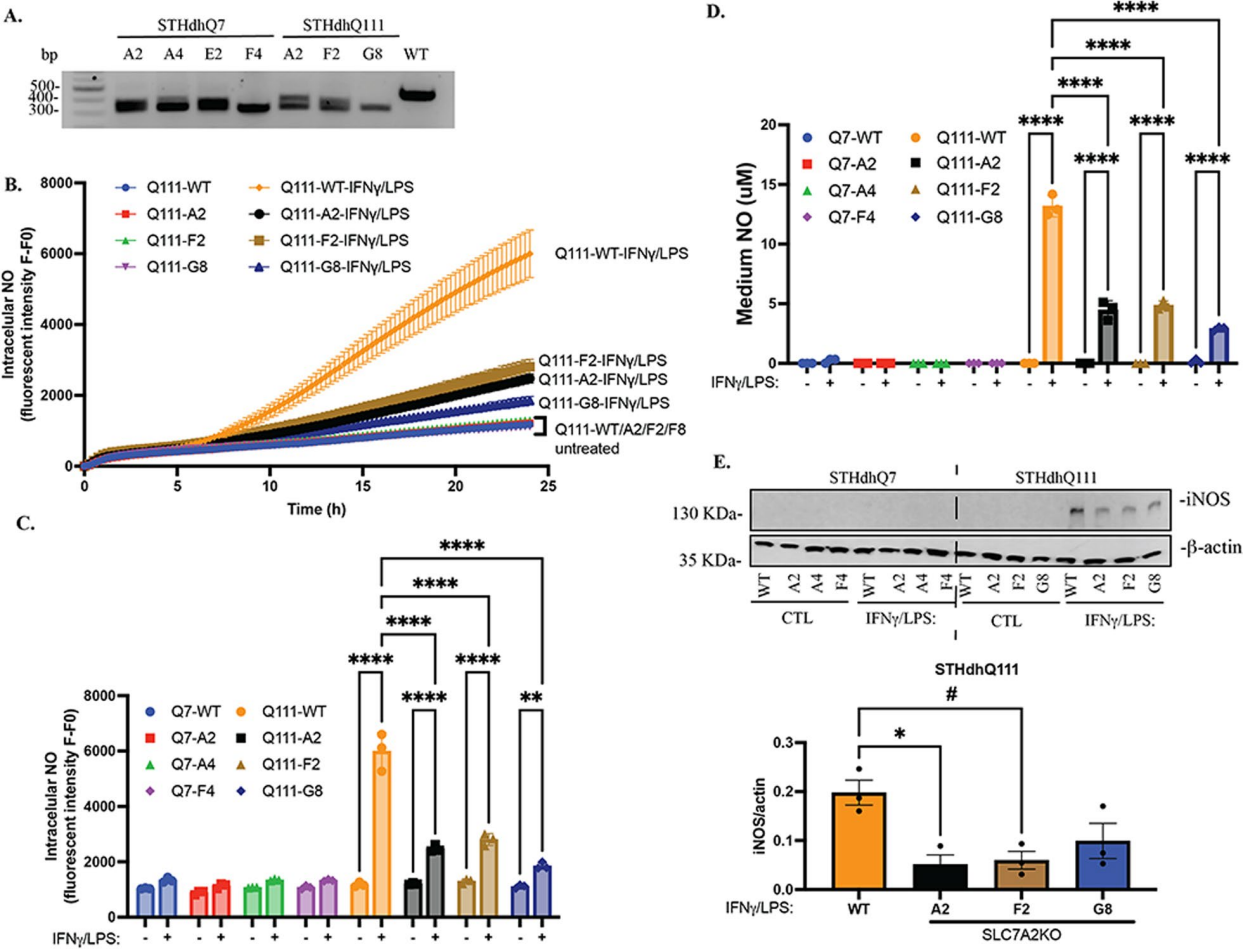
Knocking out SLC7A2 in STHdhQ111 cells attenuates neuroinflammatory responses. **A** Genotyping of SLC7A2KO clones shows a large fragment deletion in SLC7A2 PCR products compared to that in parental STHdhQ111 cells. **B** Intracellular NO production in real time in WT and SLC7A2KO cells in response to IFNγ/LPS treatment. **C** Quantification of intracellular NO levels 24 h after IFNγ/LPS treatment under different conditions as indicated. ****p < 0.0001. **D** Quantification of medium NO levels 24 h after IFNγ/LPS treatment under different conditions as indicated. **p = 0.0032, ****p < 0.0001. In **C**, **D**, two-way ANOVA followed by Tukey’s multiple comparisons test. **E** Western blot analysis of iNOS expression under different conditions as indicated. β-Actin was used as a loading control. The bottom panel shows the quantification of iNOS expression from three independent Western blots by densitometry analysis. *p = 0.016, ^#^*p* = 0.022, one-way ANOVA followed by Tukey’s multiple comparisons test

To further determine the role of SLC7A2 in this process, we knocked out SLC7A2 in STHdhQ7 and Q111 cells using the CRISPR–Cas9 genetic editing approach. A pool of three sgRNAs targeting the exon 2 region of mouse *Slc7a2* was used (Additional file 1: Fig S1A). Since we do not have a reliable SLC7A2 antibody to validate the knockout at the protein level by Western blot, we focused on clones containing large fragment deletions that can be easily identified by genotyping and DNA gel electrophoresis. We identified four SLC7A2KO clones from STHdhQ7 (Fig. 4A, A2, A4, E2 and F4) and Q111 cells (Fig. 4A, A2, F2 and G8) and verified a 118-bp fragment deletion with Sanger sequencing followed by online ICE analysis (Fig. 4A, Additional file 1: Fig. S1B). This leads to a premature stop codon within exon 2 of the coding region. We first investigated whether knocking out SLC7A2 blocks neuroinflammation-induced NO production. In this study, three independent SLC7A2KO clones from STHdhQ7 (designated Q7-A2, Q7-A4, Q7-F4) and STHdhQ111 (designated Q111-A2, Q111-F2, Q111-G8) were used. We first monitored intracellular NO levels in real time. Both parental STHdhQ7 and SLC7A2KO cells were not responsive to INFγ/LPS treatment (Fig. 4C and Additional file 1: Fig. S1C). Intriguingly, while intracellular NO levels were markedly increased in parental STHdhQ111 cells (Q111-WT) with INFγ/LPS stimulation (Figs. 2D, E, 4B, C), knocking out SLC7A2 significantly attenuated this increase (Fig. 4B, C). This is also true for medium NO production (Fig. 4D). We next measured iNOS expression by Western blotting and found that iNOS levels were significantly reduced in Q111-A2 and Q111-F2 cells in response to INFγ/LPS treatment (Fig. 4E). Q111-G8 cells also demonstrated a decrease in iNOS levels but did not reach significance (Fig. 4E, *p* = 0.10). Taken together, these data suggest that Arg transportation across the plasma membrane by SLC7A2 is pivotal to the elevated inflammatory response of STHdhQ111 cells.

### Transcriptomic changes in STHdhQ7 and Q111 cells in response to INFγ/LPS treatment

**Fig. 5:**
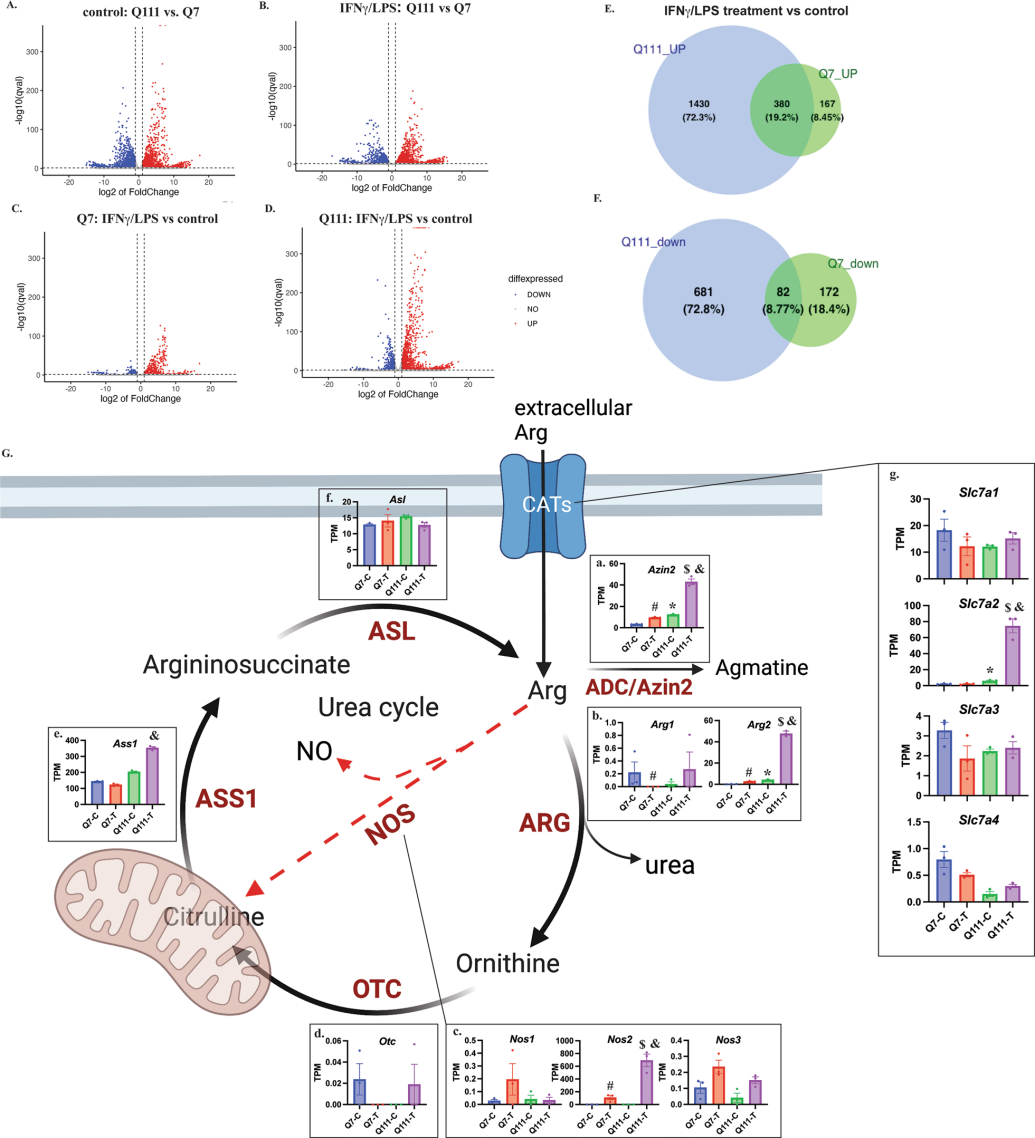
RNA-seq analysis of DEGs in STHdhQ111 and Q7 cells in response to IFNγ/LPS treatment. **A**–**D** Volcano plot showing DEGs by pairwise comparisons across different conditions as indicated. **E**, **F** Venn diagram showing upregulated (**E**) and downregulated (**F**) DEGs (treatment vs*.* control) in STHdhQ7 and Q111 cells. **G** A simplified diagram illustrating Arg biosynthesis and metabolism with key proteins indicated. Created with BioRender.com. The insets **a**–**g** are bar graphs showing the quantification of each related gene transcript in TPM across different conditions. The TPM values were extracted from RNA-seq analysis (now deposited as GSE241325). *Indicates significance between Q111-C and Q7-C; ^#^indicates significance between Q7-T and Q7-C; ^$^indicates significance between Q111-T and Q111-C; ^&^indicates significance between Q111-T and Q7-T. The significance (*q* < 0.05) was calculated using the R package DESeq2 between two different groups

To assess the global transcriptomic changes in STHdhQ7 and Q111 cells in response to INFγ/LPS challenge, we performed RNA-seq on STHdhQ7 and Q111 cells in the presence or absence of INFγ/LPS treatment. The principal component analysis (PCA) plot demonstrates that the RNA profile of biological replicates (*N* = 3) of each treatment was closely clustered together (Additional file 1: Fig. S2A). We then performed pairwise comparisons to calculate significant changes in the transcriptome across different conditions. The volcano plot indicates that there is a large number of differentially expressed genes (DEGs) between STHdhQ111 and Q7 cells in control and after IFNγ/LPS challenge (Fig. 5A, B, |log2(foldchange)| > 1 and *q* < 0.05). Among these DEGs, the top KEGG pathways were related to inflammation in both STHdhQ7 and Q111 cells (Additional file 1: Fig. S2B, C), indicating that both cell types are responsive to neuroinflammatory challenges. This is consistent with the role of INFγ/LPS as a strong inducer of inflammation. However, the levels of activation are different between the two cell types. In response to INFγ/LPS treatment, STHdhQ111 cells demonstrated a more robust expression profile change than STHdhQ7 cells (Fig. 5C vs. D). This is also clearly demonstrated by the Venn diagram showing that more genes were either up- or down-regulated in STHdhQ111 cells than in Q7 cells (Fig. 5E, F).

Arg is one of the most metabolically versatile amino acids. The homeostasis of intracellular Arg is maintained by Arg transporters via CATs, protein breakdown/recycle, Arg biosynthesis and metabolism. In the present study, we specifically examined the transcriptomic changes in these immediate key active genes related to Arg biosynthesis and degradation. As illustrated in Fig. 5G, Arg is converted to ornithine, citrulline and agmantine by arginase (ARG, including ARG1 and ARG2), nitric oxide synthase (NOS, including nNOS, iNOS and eNOS) and arginine decarboxylase (ADC/Azin2), respectively. Ornithine can then be converted to citrulline by ornithine transcarbamylase (OTC). Through a two-step reaction catalyzed by arginosuccinate synthase 1 (ASS1) and arginosuccinate lyase (ASL), citrulline is then converted back to Arg. The insets in Fig. 5G show the TPM counts of the respective mRNAs in each cell type under control (Q7-C, Q111-C) and INFγ/LPS treatment (Q7-T, Q111-T). In the nontreated control group, *Azin2* and *Arg2* mRNAs were significantly upregulated in STHdhQ111 cells (Fig. 5G, insets a–b, *Q111-C vs. Q7-C). In response to INFγ/LPS treatment, *Azin2*, *Arg2* and *Nos2* mRNAs were significantly upregulated in both STHdhQ7 and Q111 cells (Fig. 5G, insets a–c, ^#^Q7-T vs. Q7-C; ^$^Q111-T vs. Q111-C). Notably, the increases in *Arg2* and *Azin2* mRNAs were similar in STHdhQ111 and Q7 cells (*Arg2*: 11.92-fold increase in Q111 cells compared to 14.91-fold increase in Q7 cells; *Azin2*: 3.55-fold increase in Q111 cells compared to 2.95-fold increase in Q7 cells). However, *Nos2*, which encodes iNOS, was significantly upregulated in Q111 cells (457.13-fold increase) compared to STHdhQ7 cells (74.32-fold increase), which is consistent with our qRT-PCR analysis (Fig. 2C). Moreover, *Arg2, Ass1, Azin2* and *Nos2* were significantly upregulated in Q111 cells compared to Q7 cells after INFγ/LPS treatment (Fig. 5G, insets a–c, e, and Q111-T vs, Q7-T), indicating a more dynamic Arg metabolism in challenged HD cells.

Finally, we examined the transcriptomic changes in genes regulating Arg transport, namely, *Slc7a1-a4*. Only *Slc7a2* mRNA was significantly upregulated in STHdhQ111 cells under control conditions (Fig. 5G, inset g). Importantly, INFγ/LPS treatment led to a more robust increase in *Slc7a2* mRNA levels in STHdhQ111 cells compared to Q7 cells. As a result, there was an ~ 32-fold increase in *Slc7a2* mRNA in INFγ/LPS-treated STHdhQ111 cells compared to Q7 cells (Fig. 5G, inset g).

### Arginine metabolism in response to INFγ/LPS treatment

**Fig. 6:**
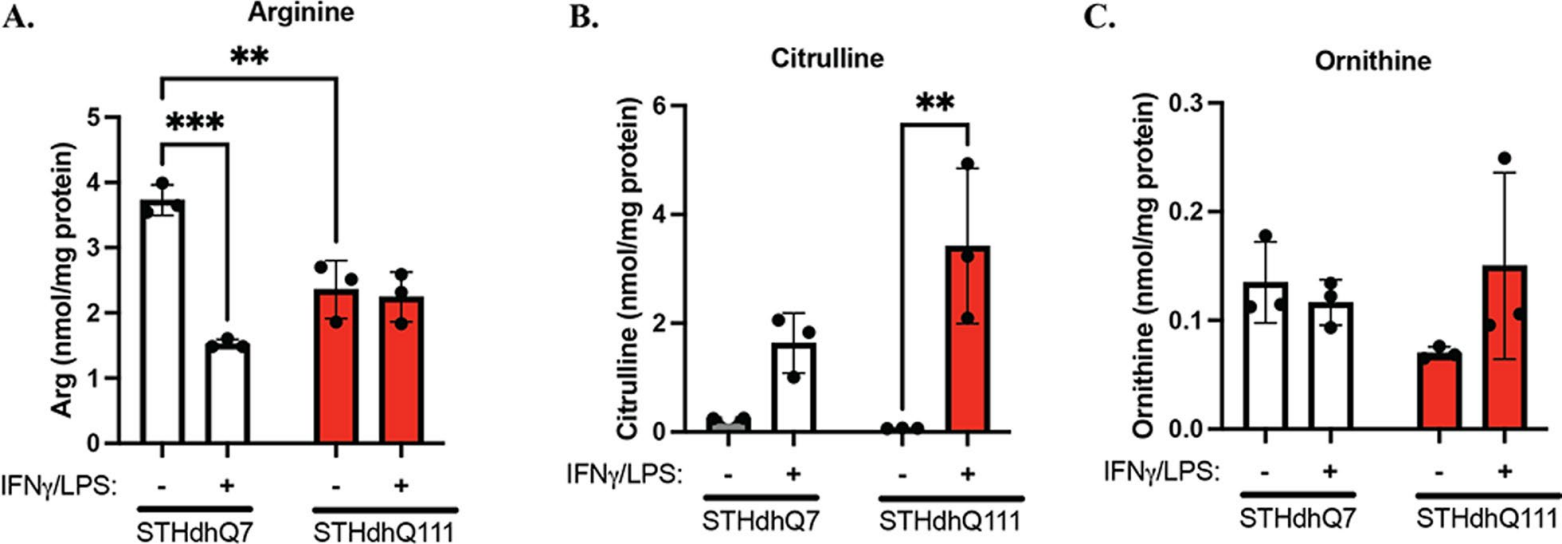
Arg metabolism in STH cells in response to IFNγ/LPS treatment. HPLC–MS/MS analysis of Arg (**A**, **p = 0.0033, ***p = 0.001), citrulline (**B**, **p = 0.0030) and ornithine (**C**) levels in STHdhQ7 and Q111 cells in the presence or absence of IFNγ/LPS treatment. *N* = 3 independent samples under each condition. In **A**–**C**, two-way ANOVA followed by Tukey’s multiple comparisons test

To further investigate changes in Arg metabolism in STHdhQ7 and Q111 cells in response to INFγ/LPS treatment, we measured the levels of intracellular Arg (Fig. 6A) and its major metabolites, citrulline (Fig. 6B) and ornithine (Fig. 6C), by HPLC–MS/MS analysis. A two-way ANOVA on Arg levels revealed that there was a significant interaction between cell type (Q7 vs*.* Q111) and treatment (control vs*.* INFγ/LPS) [Fig. 6A, *F*(1,8) = 32.42, *p* = 0.0005]. Surprisingly, the Arg level in STHdhQ111 cells was significantly lower than that in Q7 cells under control conditions, as revealed by post hoc Tukey analysis (Fig. 6A, p = 0.0033). It is possible that Arg metabolism is more active in STHdhQ111 cells, thus reducing free Arg levels. This deduction is based on the RNA-seq analysis of relevant enzymes involved in Arg metabolism (Fig. 5G). Interestingly, INFγ/LPS treatment significantly decreased Arg levels in STHdhQ7 cells (Fig. 6A, *p* = 0.0001) but not in STHdhQ111 cells (Fig. 6A, *p* = 0.97). In regard to citrulline and ornithine levels, two-way ANOVA did not reveal a significant interaction between cell type and treatment [Fig. 6B, *F*(1, 8) = 5.004, *p* = 0.055; Fig. 6C, *F*(1,8) = 3.177, *p* = 0.112]. However, there was a significant effect of the neuroinflammatory challenge on citrulline levels [Fig. 6B, *F*(1, 8) = 28.60, *p* = 0.0007]. After INFγ/LPS treatment, there was a significant increase (~ 50-fold) in citrulline levels in STHdhQ111 cells (Fig. 6B, **p = 0.003). In contrast, citrulline levels increased by ~ sixfold in STHdhQ7 cells (Fig. 6B, *p* = 0.20). The sharp difference in citrulline levels in response to INFγ/LPS treatment is consistent with the NO measurements, as shown in Fig. 2D, E. Additionally, there were no significant changes in ornithine levels in STHdhQ7 and Q111 cells before and after INFγ/LPS treatment, indicating that the arginase pathway is not the major route of Arg metabolism under neuroinflammation (Fig. 6C). Taken together, these data indicate that the conversion of Arg to citrulline and NO by iNOS is the major pathway for Arg metabolism in STHdhQ7 and STHdhQ111 cells under neuroinflammation.

### Protein S-nitrosylation was increased in STHdhQ111 cells after INFγ/LPS treatment

**Fig. 7:**
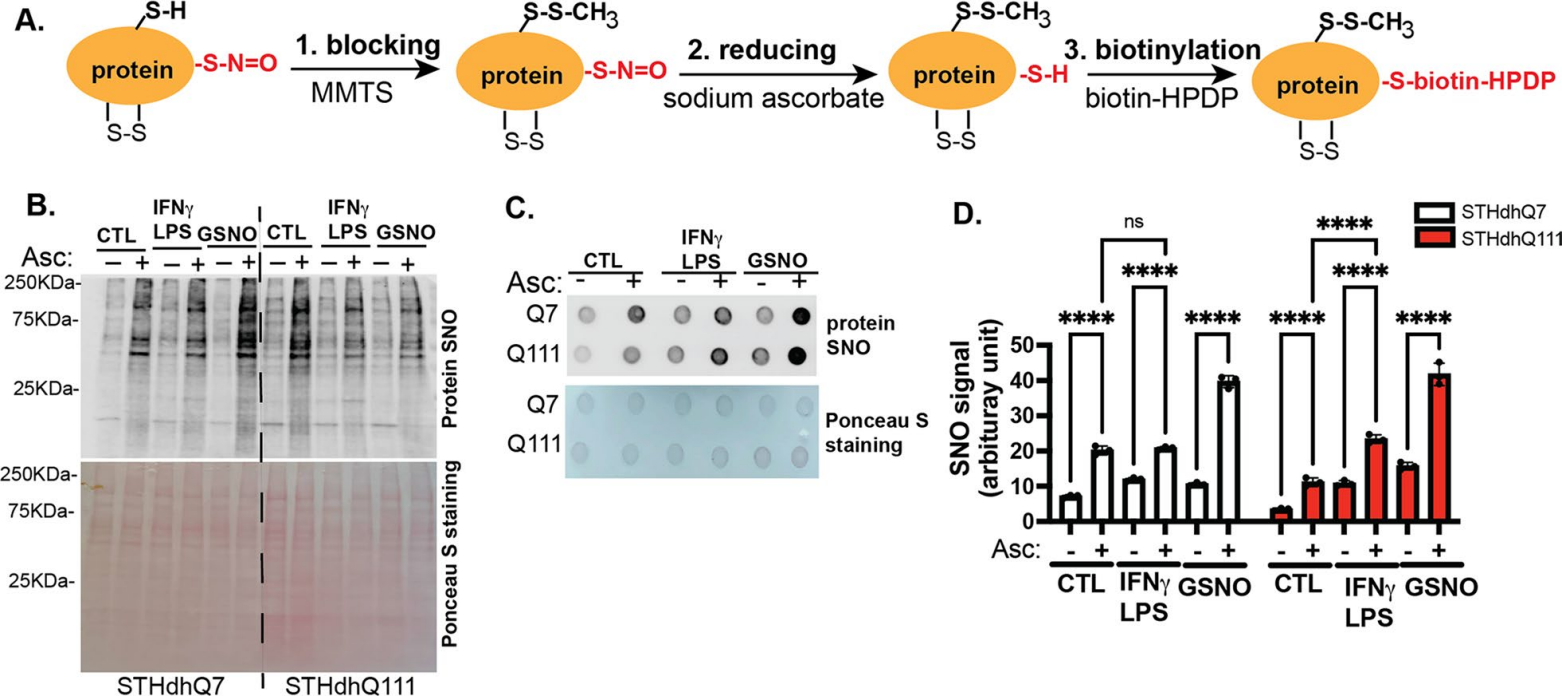
Protein SNO is increased in STHdhQ111 cells in response to IFNγ/LPS treatment. **A** A simplified flowchart depicting the three steps in the biotin switch assay. **B** Representative Western blot analysis of nitrosylated protein from the biotin switch assay. **C** Representative dot blot analysis of nitrosylated protein from the biotin switch assay. In **B**, **C**, nitrosylated protein was detected by streptavidin labeling (upper image). The bottom image shows the total protein by Ponceau S staining. **D** Quantification of nitrosylated protein from three independent dot blots. ****p < 0.0001. Two-way ANOVA followed by Tukey’s multiple comparisons test

We next asked whether SNO-proteins were increased in HD cells in response to abnormally high levels of NO during neuroinflammation using the three-step biotin switch assay [18, 29] as outlined in Fig. 7A: (1) blocking free Cys by MMTS; (2) reducing nitrosylated Cys to free Cys by sodium ascorbate; and (3) labeling reduced Cys by biotin-HDPD. In the absence of sodium ascorbate (Asc), nitrosylated cysteine cannot be reduced and labeled with biotin, thus serving as an internal control for the assay. The resulting biotinylated SNO-proteins were further detected using fluorescently labeled streptavidin in Western blot and dot blot analyses (Fig. 7B, C). The dot blot assay was used for quantification in Fig. 7D. The signals for biotinylation were significantly increased in the presence of Asc compared to the signals in the absence of Asc, indicating the specific recognition of SNO proteins by Asc. INFγ/LPS treatment further increased SNO proteins compared to the control in STHdhQ111 but not Q7 cells. Incubation with S-nitrosoglutathione (GSNO), an NO donor, caused significant increases in SNO levels in both cell types, serving as a positive control for the assay. Together, these data suggest that nitrosylated proteins were increased in STHdhQ111 cells in response to neuroinflammation.

### Mitochondrial fission was increased in STHdhQ111 cells after INFγ/LPS treatment

**Fig. 8:**
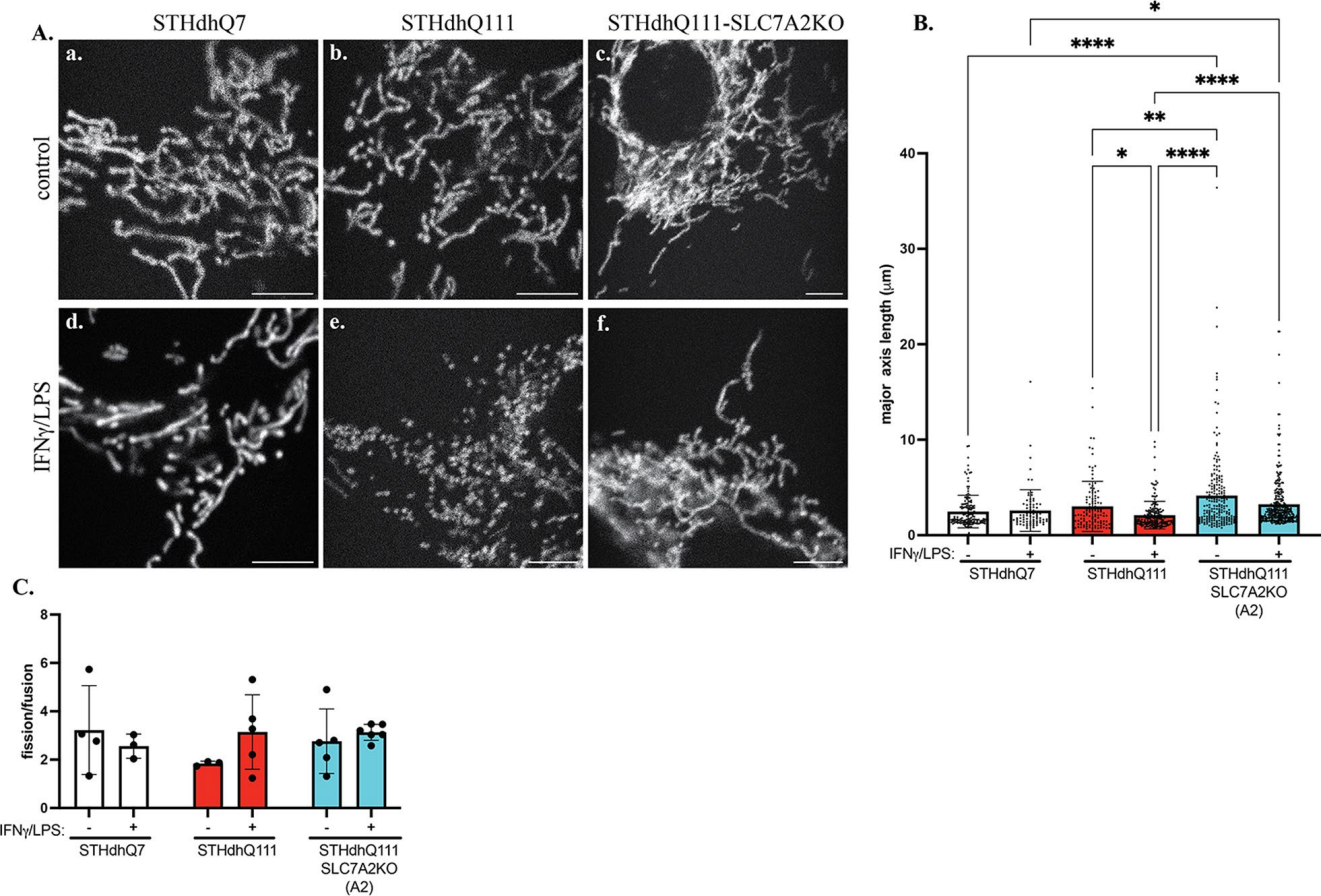
More fragmented mitochondria in STHdhQ111 cells in response to IFNγ/LPS treatment. **A** Representative frames extracted from time-lapse images showing changes in mitochondrial morphology in STHdhQ7-, STHdhQ111- and STHdhQ111-SLC7A2KO cells under control conditions (**a**–**c**) and 24 h after neuroinflammation (**d**, **e**). Scale bar: 5 μm. **B** Quantification of individual mitochondrial length from time-lapse images in different groups as indicated. Data represented as median length with interquartile range. *p < 0.05, **p < 0.01, ***p < 0.001, ****p < 0.0001. One-way ANOVA followed by Kruskal–Wallis multiple comparisons test. **C** Quantification of mitochondrial fission/fusion events in different groups as indicated. In **C**, *N* = 4–6 cells per condition. Two-way ANOVA followed by Tukey’s multiple comparisons test

It has been reported that elevated NO levels increase SNO-Drp1 levels, which activate its enzymatic activity, leading to increased mitochondrial fission in HD neurons [22]. We thus investigated the functional implication of neuroinflammation-induced abnormal NO production on mitochondrial fission. We labeled mitochondria with mito-TurboRFP and analyzed changes in mitochondrial morphology in response to neuroinflammation. Twenty-four hours after INFγ/LPS treatment, the length of mitochondria was clearly shorter in STHdhQ111 cells than in their respective controls in paraformaldehyde-fixed cells (Additional file 1: Fig. S3), suggesting that there was more fission activity in STHdhQ111 cells during the neuroinflammatory challenge. Interestingly, adding the iNOS inhibitor 1400W hydrochloride appeared to prevent fission (Additional file 1: Fig. S3). We further performed live-cell imaging to monitor mitochondrial dynamics in STHdhQ7, Q111 and Q111-SLC7A2KO cells under control conditions and 24 h after neuroinflammatory challenge. Individual mitochondrial length was then measured and quantified using Mitometer, a MATLAB application for unbiased, automated mitochondrial segmentation, tracking and analysis of mitochondrial morphology and movements [35]. The analysis of individual mitochondrial length is presented in Fig. 8B. There were no significant changes in mitochondrial length between control and INFγ/LPS-treated STHdhQ7 cells (Fig. 8A—a, c, B). In contrast, a significant decrease in mitochondrial length in challenged STHdhQ111 cells compared to the control was noted (Fig. 8A—b, e, B). Knocking out SLC7A2 prevented abnormal mitochondrial fission in STHdhQ111 cells as indicated by no significant changes of mitochondrial length between challenged and control STHdhQ111-SLC7A2KO cells (Fig. 8A—c, f, B). There was a trend for increased fission events in challenged STHdhQ111 cells (Fig. 8C). It is possible that the dynamic changes of fission/fusion may have occurred at an earlier time point during neuroinflammation. It is conceivable that a temporal analysis of fission/fusion along inflammatory challenge would be useful assessing the dynamic changes.

### Dietary Arg supplements may accelerate HD progression in human patients

Neuroinflammation is frequently observed in HD patients at the early stage [48]. Arg is found in most protein-enriched foods and is also available as a supplement. Based on the molecular studies here, we wondered whether Arg supplementation hastens HD progression in patients. Using the Enroll-HD PDS5 patient dataset, we filtered individuals taking Arg supplements and compared their disease progression in terms of motor score, TFC score and MMSE score recorded from their multiple visits to those who did not take the supplements. Cysteine (Cys) has been shown to be beneficial for HD patients [49, 50], we therefore included Cys supplements in the analysis for comparison. This initial filtering has an unbalanced sample size with only 24 patients taking Arg supplements and 57 patients taking Cys supplements compared to > 20K patients taking other supplements or having no information available (Ctl group). We then used propensity score matching as detailed in the method to estimate the average marginal effect of Arg supplement on disease progression accounting for confounding by the included covariates. After matching with age, CAG repeat, sex and race, all standardized mean differences for the covariates were below 0.001 and all standardized mean differences for squares and two-way interactions between covariates were below 0.01, indicating adequate balance.

**Fig. 9:**
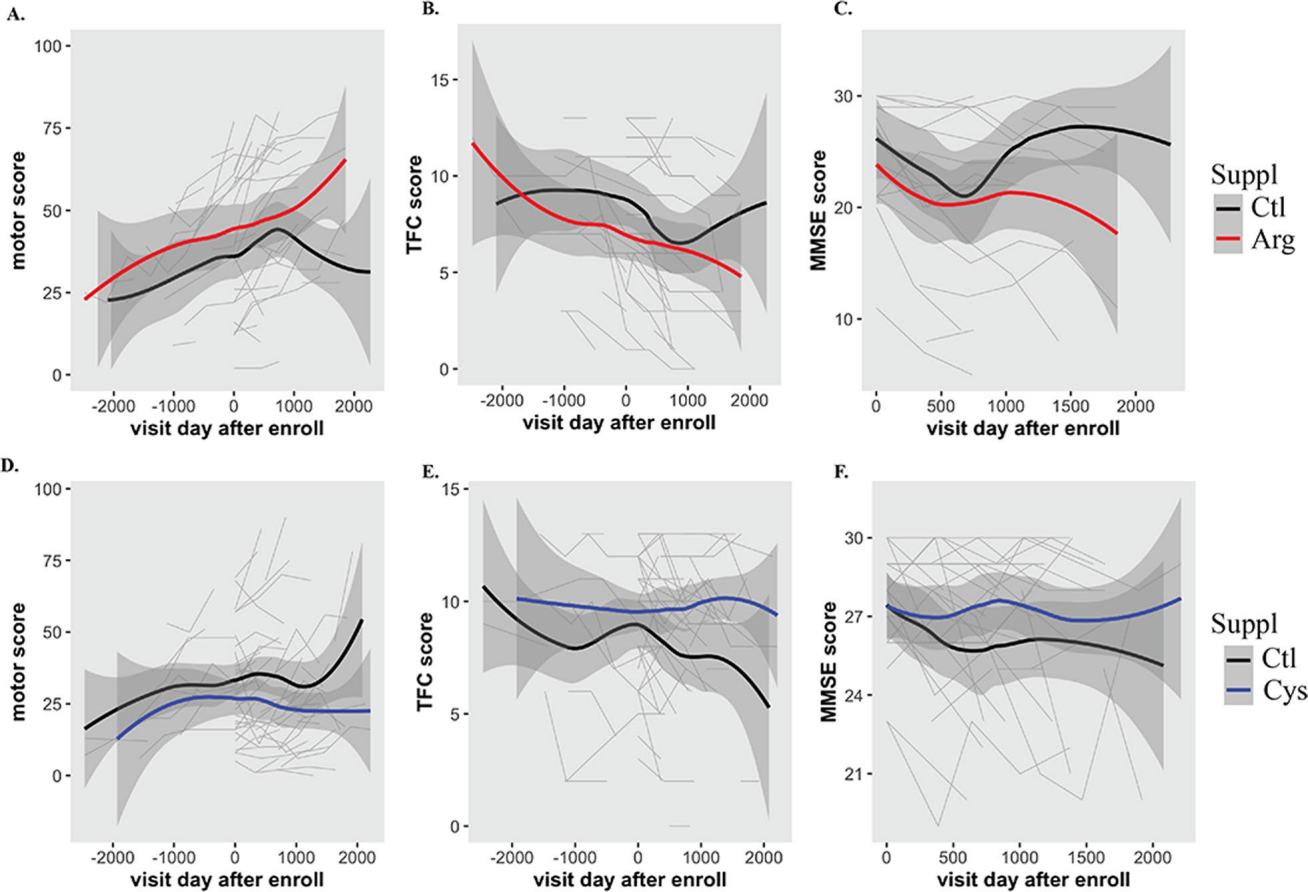
Enroll-HD PDS5 dataset analysis. **A**–**C** Temporal changes in motor (**A**), TFC (**B**) and MMSE (**C**) scores in patients taking Arg supplements, compared to others not taking Arg and Cys (Ctl). **D**–**F** Temporal changes in motor (**D**), TFC (**E**) and MMSE (**F**) scores in patients taking Cys supplements, compared to others not taking Arg and Cys (Ctl). The gray backgrounds are lines tracking score changes from individual patients. The regression line was calculated using geom_smooth() from the R package

The matching dataset for Arg vs Ctl group contains 17 HD patients taking Arg supplements and 59 control subjects. The matching dataset for Cys vs Ctl group contains 42 HD patients taking Cys supplements and 80 control subjects. Using the matched datasets, we found that patients taking Arg supplements generally exhibited accelerated disease progression than patients who did not take the supplement, which was reflected in a quicker decline in motor function (Fig. 9A), TFC (Fig. 9B) and MMSE scores (Fig. 9C). Interestingly, cysteine supplements delayed disease progression (Fig. 9D–F). The estimated effect for Arg supplement on motor score was 7.10 (SE = 2.64, *p* = 0.007), on TFC score was − 1.28 (SE = 0.47, *p* = 0.006), indicating that the average effect of Arg supplement for HD patients who took them can be harmful. On the contrary, the estimated effect for Cys supplement on motor score was − 9.61 (SE = 2.00, *p* = 1.55 × 10^–6^), on TFC score was 2.11 (SE = 0.35, *p* = 1.25 × 10^–9^), indicating a beneficial effect for HD patients taking Cys supplements. The estimation on either Arg or Cys effect on MMSE score failed to yield any results using the same fit model. It is important to mention that the duration and dosage of the supplements varies among patients. Moreover, most patients also take multiple other supplements. It is therefore difficult to estimate the significance of the differences. Although a larger cohort of patients are needed for validation, this analysis suggests that dietary Arg supplementation may accelerate disease progression, while Cys supplementation can slow disease progression.

## Discussion

In this study, we provide the first line of evidence for the selective upregulation of SLC7A2/CAT2 compared to other arginine transporter family proteins (SLC7A1, A3 and A4) in HD cellular models and patients. Consequently, our study unveils the abnormally high production of iNOS and NO in response to neuroinflammatory challenges in HD cells, leading to increased nitrosative stress and protein nitrosylation. Importantly, knocking out SLC7A2 significantly blocked the elevated inflammatory response in HD cells. Our study was mainly performed using STHdhQ7 and Q111 striatal cell lines. While it has been noted that modified morphological and proliferation properties are inherent characteristics and potential confounding factors in the STHdh cell model of HD [51], we have validated our findings by extending our investigation to include the YAC128 HD mouse model. Moreover, to mitigate the potential confounding effects arising from morphological differences between the STHdhQ7 and Q111 cell lines, we opted for a strategic approach. Instead of directly comparing stress-induced changes between STHdhQ7 and Q111, our experimental design and data interpretation focus on discerning stress-related alterations within the same cell type. Subsequently, we compare these changes across the two cell lines. Our studies thus reveal a novel role of SLC7A2 in exacerbating neuroinflammation in HD and accelerating HD progression.

We demonstrate that muHTT upregulates SLC7A2 mRNA transcription in several HD cellular models and HD patients. Transcriptional dysregulation has been proposed as an important pathogenic mechanism in HD [4, 5]. The underlying mechanism deserves further investigation. The transcriptional role of HTT/muHTT can be attributed to altered protein–protein interactions between HTT and transcription factors (TFs) or histone-modifying enzymes. A number of TFs that have altered interactions with muHTT have been identified, including but not limited to p53 and CREB-binding protein (CBP) [52], NeuroD [53], methyl-CpG binding protein 2 (MeCP2) [54], REST [55], SP1 and its coactivator TAFII130 [56]. It is thus possible that muHTT affects the TFs that are critical in regulating *SLC7A2* transcription. By examining the gene targets of several known TFs using the ENCODE Transcription Factor Target Dataset from Harmonizome [57], we found that SLC7A2 is the gene target of REST, SP1 and E2F1, which have all been implicated in HD [3]. An important feature of SLC7A2 is that it is inducible in response to inflammation compared to the constitutive expression of SLC7A1. This is well documented in macrophages [23, 24]. For example, it has been reported that TNFα causes increased expression of the SLC7A2/CAT2B gene, while SLC7A1/CAT1 expression is not altered in human endothelial cells [58]. In the brain, SLC7A2 expression was increased in primary mouse astrocytes treated with INFγ/LPS [59]. Here, we showed that SLC7A2 mRNA was increased in STHdhQ111 cells in response to neuroinflammatory challenge by RNA-seq analysis (Fig. 5G), indicating that muHTT may further upregulate *SLC7A2* mRNA expression during neuroinflammation.

An important observation from the current study is that extracellular Arg translationally regulates iNOS induction in response to neuroinflammatory stimulation (Fig. 3). This is consistent with a previous study reporting that extracellular Arg regulates iNOS expression at the translational level in primary rat astrocytes in response to cAMP and IFNγ challenge [47]. Mechanistically, depletion of Arg increased the phosphorylation of eukaryotic initiation factor-2α (eIF2α), which in turn inhibited iNOS translation [47]. Together, these findings help to explain the Arg paradox, which refers to the phenomenon that NO production depends on the extracellular Arg concentration even though NOS is theoretically saturated with intracellular Arg [60]. Our study further suggests the pivotal role of SLC7A2 in translationally upregulating iNOS production during neuroinflammation. This is supported by the observation that knocking out SLC7A2 in STHdhQ111 cells significantly reduced iNOS induction and NO production in response to INFγ/LPS treatment. In line with our observation, it was reported that iNOS and NO production was greatly impaired in primary mouse SLC7A2KO astrocytes challenged with INFγ and LPS [59]. Arg uptake during the challenge was also significantly reduced [59]. Furthermore, SLC7A2KO mice exhibited a block of hyperoxia-induced increase in exhaled NO production [61] and a downregulation in Arg transport in response to Th1- and Th2-type cytokines [23]. Therefore, compared to other Arg transporters, SLC7A2 is critical in mediating Arg uptake and neuroinflammatory responses.

One downstream effect of supraphysiological NO production is nitrosative stress, which causes NO-mediated aberrant protein nitrosylation. Aberrant protein SNO can initiate pathological signaling cascades and is implicated in neurodegenerative diseases [18–21, 62]. In the brain, proteomic analyses revealed that many synaptic proteins were nitrosylated in AD [63, 64], chronic stress [65] and aging [66]. In Drosophila and zebrafish models of HD, nitrosative stress inhibited autophagosome formation through aberrant nitrosylation of JNK1 (c-Jun N-terminal protein kinase 1) and IKKβ (IkappaB kinase beta), while inhibition of NOS activity promoted autophagy and reduced mutant HTT aggregation and neurodegeneration [67]. Furthermore, nitrosative stress, as reflected by increased plasma levels of nitrotyrosine in HD patients, has been reported [68]. It was previously reported that expression of N-terminal muHTT in primary rat cortical neurons increased nitrosylation and activation of Drp-1, leading to increased mitochondrial fission [22]. Moreover, Drp-1 nitrosylation was increased in brain lysates prepared from BACHD transgenic mice and cortex regions of postmortem HD patients [22]. Consistently, we showed that protein nitrosylation is elevated in HD cells upon neuroinflammatory insult and that more mitochondria in STHdhQ111 cells were fragmented after neuroinflammation (Fig. 8). To further support our finding, increased Drp-1 dependent mitochondrial fragmentation was also reported in R6/1 striatal culture [69]. Increased mitochondrial fragmentation was associated with decreased ER–mitochondria contact sites in the striatum of R6/1 mice, knock-in mutant Hdh^Q7/Q111^ mice (the same transgenic mice where the STHdhQ7 and Q111 cell lines used in this study were derived from) and postmortem human brain [69]. Therefore, the notion of increased nitrosylation of DRP-1 could contribute to the pathogenesis of HD is in line with our observations. Since neuroinflammation is frequently observed in HD patients, it is important to further identify aberrantly nitrosylated proteins through proteomic approaches and study their impacts on HD pathogenesis and progression.

Arg plays an important role in various physiological processes [8]. Importantly, Arg is also a key regulator of the mTORC1 pathway [70]. Therefore, Arg has become a popular supplement in the US and has been marketed online as a beneficial agent for immunological disorders and general health. However, its use in clinically ill patients is controversial [71]. Our molecular studies raise the potential for the use of Arg supplements in HD patients. Indeed, by analyzing the Enroll-HD patient dataset, we found that Arg supplementation may accelerate HD progression (Fig. 9). Although this finding can be further confounded by the complexity of diet intake and other uncontrollable factors across the patient cohort, animal studies from R6/1 mice appear to support this observation. Deckel et al. showed that a high-Arg diet accelerated HD progression in R6/1 mice [72]. In their study, mouse chow with 0%, 1.2% (typical mouse chow) and 5% Arg was supplied to 12-week-old control and R6/1 mice for 10 weeks. The 5% Arg diets in HD mice significantly accelerated the time of onset of body weight loss, motor impairments and increased resting cerebral blood flow (CBF) in HD relative to the control. Due to the essential role of Arg, the HD group with a 0% Arg diet did not show improvement in motor testing. The underlying molecular basis was linked to NO production [72].

On the other hand, beneficial effects of Arg supplements in treating polyQ diseases, including mouse models of SCA1 (spinocerebellar ataxia type 1) and SBMA (spinal and bulbar muscular atrophy) [73] and a Drosophila model of HD [74], have been reported. In these studies, Arg was reported to function as a chemical chaperone that prevents polyQ aggregate formation in vitro*,* and Arg supplementation could improve animal behaviors at the early stages of SCA1 and SBMA. However, the metabolism of Arg is not considered, and the long-term effect of Arg supplementation is not clear. We cannot preclude the possibility that Arg prevents muHTT aggregate formation. However, it remains unsettled whether the soluble, oligomeric, or aggregated form of muHTT is more toxic. Our present study is mechanistically distinct in that we examined the effect of NO, one of the major Arg metabolites, under neuroinflammation. In line with this, it has been reported that Arg depletion improves spinal cord injury via immunomodulation and reduced NO production [75]. A high-Arg diet accelerated HD progression in R6/1 HD mice, possibly through increased NO production [72]. Clearly, there are questions regarding the role of Arg supplements in treating neurological diseases. Furthermore, we did not observe differences in *SLC7A2* mRNA expression between WT and SCA1 mice after the examination of the SCA1-related RNA-seq datasets GSE163885 [76] and GSE122099 [77]. It is likely that Arg supplements may potentially pose a greater risk to HD patients due to the specific upregulation of SLC7A2 expression. We therefore believe that the application of Arg in the HD clinical regimen is still premature and requires further investigation from multiple perspectives.

Last, it is important to mention that Arg metabolism is complex due to the expression of multiple enzymes that utilize Arg as the substrate. Consequently, the different biologically active products from Arg metabolism may also participate in HD pathogenesis, which needs further investigation.

## Conclusions

In summary, this study presents a model of how SLC7A2 may affect HD progression during neuroinflammation. The clinical relevance of these findings warrants further studies in a larger cohort of HD patients.

### Supplementary Information

**Additional file 1: Figure S1.** Knocking out SLC7A2 by the CRISPR–Cas9 system. **A** Scheme showing the three sgRNA sequence targeting exon 2 of the mouse *Slc7a2* gene (pink, guide 1, 2 and 3), the PCR primers for amplification (green, forward and revers primers) and the sequencing primers for Sanger sequencing (orange, sequencing primer). **B** Confirmation of the large fragment deletion in the SLC7A2KO clones in STHdhQ7 and Q111 cells with Sanger sequencing followed by ICE analysis using the online tool from Synthego. **C** Intracellular NO levels monitored in real-time using DAF-2DA in STHdhQ7, Q7-SLC7A2KOs, Q111 and Q111-SLC7A2KO in the presence or absence of IFNg/LPS treatment. **Figure S2.** RNA-seq analysis of transcriptomic changes in STHdhQ7 and Q111 cells in response to IFNg/LPS treatment. **A** PCA plot of all the samples used in the RNA-seq analysis. Q7-C: STHdhQ7 untreated; Q7-C: STHdhQ7 cells with 24-h IFNg/LPS treatment; Q111-C: STHdhQ111 untreated; Q111-C: STHdhQ111 cells with 24-h IFNg/LPS treatment. **B**, **C** KEGG pathway enrichment analysis in STHdhQ7 (**B**) and Q111 (**C**) cells in response to IFNg/LPS treatment. **Figure S3.** Mitochondrial fragmentation in STHdhQ111 cells in response to IFNg/LPS treatment. STHdhQ7 and Q111 cells were transfected with mito-turboRFP and treated with IFNg/LPS for 24 h in the presence or absence of 1400W hydrochloride. Cells were then fixed in 4% paraformaldehyde with 4% sucrose to preserve mitochondrial morphology. Images were taken with a 60× oil objective (CFI Plan Apochromat Lambda 60× Oil, numerical aperture = 1.4) using a laser scanning confocal microscope (Nikon A1R). Scale bar: 5 μm.

## Data Availability

RNA-seq data were deposited into the GEO database under accession number GSE241325 and are available at the following URL: https://www.ncbi.nlm.nih.gov/geo/query/acc.cgi?acc=GSE241325. All other data supporting the findings of this study are available within the paper and its Additional file.

## Abbreviations

Arg: Arginine
Biotin-HPDP: *N*-[6-(Biotinamido)hexyl]-3′-(2′-pyridyldithio)propionamide
CAT: Cationic amino acid transporter
CRISPR–Cas9: Clustered regularly interspaced palindromic repeats–CRISPR-associated protein 9
Cys: Cysteine
DAF-2 DA: 4,5-Diaminofluorescein diacetate
DEG: Differentially expressed genes
DMEM: Dulbecco’s modified Eagle medium
DRP-1: Dynamin related protein-1
GSNO: *S*-Nitrosoglutathione
HD: Huntington’s disease
HTT: Huntingtin
ICE: Inference of CRISPR edits online analysis tool
IFNγ: Interferon-gamma
iNOS: Inducible nitric oxide synthase
LPS: Lipopolysaccharide
MMTS: Methyl methanethiosulfonate
MMSE: Mini-Mental State Exam
muHTT: Mutant huntingtin
NO: Nitric oxide
NOS: Nitric oxide synthase
PDS5: Periodic dataset 5
RNP: Ribonucleoprotein
RNS: Reactive nitrogen species
qRT-PCR: Quantitative real-time reverse transcription-polymerase chain reaction
SLC7A2: Solute carrier family 7 member 2
sgRNA: Single guide RNA
SNO: Protein S-nitrosylation
TBS: Tris-buffered saline
TF: Transcription factor
TFC: Total functional score

## References

[1] Group THsDCR (1993). A novel gene containing a trinucleotide repeat that is expanded and unstable on Huntington’s disease chromosomes. Cell.

[2] Saudou F, Humbert S (2016). The biology of Huntingtin. Neuron.

[3] Benn CL, Sun T, Sadri-Vakili G, McFarland KN, DiRocco DP, Yohrling GJ (2008). Huntingtin modulates transcription, occupies gene promoters in vivo, and binds directly to DNA in a polyglutamine-dependent manner. J Neurosci.

[4] Cha JH (2007). Transcriptional signatures in Huntington’s disease. Prog Neurobiol.

[5] Malla B, Guo X, Senger G, Chasapopoulou Z, Yildirim F (2021). A systematic review of transcriptional dysregulation in Huntington’s disease studied by RNA sequencing. Front Genet.

[6] Bensalel J, Xu H, Lu ML, Capobianco E, Wei J (2021). RNA-seq analysis reveals significant transcriptome changes in huntingtin-null human neuroblastoma cells. BMC Med Genom.

[7] Marti ILAA, Reith W (2021). Arginine-dependent immune responses. Cell Mol Life Sci.

[8] Tong BC, Barbul A (2004). Cellular and physiological effects of arginine. Mini Rev Med Chem.

[9] Morris SM (2006). Arginine: beyond protein. Am J Clin Nutr.

[10] Cinelli MA, Do HT, Miley GP, Silverman RB (2020). Inducible nitric oxide synthase: regulation, structure, and inhibition. Med Res Rev.

[11] Lundberg JO, Weitzberg E (2022). Nitric oxide signaling in health and disease. Cell.

[12] Martínez-Ruiz A, Lamas S (2009). Two decades of new concepts in nitric oxide signaling: from the discovery of a gas messenger to the mediation of nonenzymatic posttranslational modifications. IUBMB Life.

[13] Ahern G (2002). cGMP and S-nitrosylation: two routes for modulation of neuronal excitability by NO. Trends Neurosci.

[14] Steinert JR, Chernova T, Forsythe ID (2010). Nitric oxide signaling in brain function, dysfunction, and dementia. Neuroscientist.

[15] Hess DT, Matsumoto A, Kim S-O, Marshall HE, Stamler JS (2005). Protein S-nitrosylation: purview and parameters. Nat Rev Mol Cell Biol.

[16] Nakamura T, Oh CK, Zhang X, Tannenbaum SR, Lipton SA (2021). Protein transnitrosylation signaling networks contribute to inflammaging and neurodegenerative disorders. Antioxid Redox Signal.

[17] Choi YB, Tenneti L, Le DA, Ortiz J, Bai G, Chen HS (2000). Molecular basis of NMDA receptor-coupled ion channel modulation by S-nitrosylation. Nat Neurosci.

[18] Jaffrey SR, Erdjument-Bromage H, Ferris CD, Tempst P, Snyder SH (2001). Protein S-nitrosylation: a physiological signal for neuronal nitric oxide. Nat Cell Biol.

[19] Nakamura T, Prikhodko OA, Pirie E, Nagar S, Akhtar MW, Oh C-K (2015). Aberrant protein S-nitrosylation contributes to the pathophysiology of neurodegenerative diseases. Neurobiol Dis.

[20] Sircar E, Rai SR, Wilson MA, Schlossmacher MG, Sengupta R (2021). Neurodegeneration: impact of S-nitrosylated Parkin, DJ-1 and PINK1 on the pathogenesis of Parkinson’s disease. Arch Biochem Biophys.

[21] Pirie E, Oh CK, Zhang X, Han X, Cieplak P, Scott HR (2021). S-nitrosylated TDP-43 triggers aggregation, cell-to-cell spread, and neurotoxicity in hiPSCs and in vivo models of ALS/FTD. Proc Natl Acad Sci USA.

[22] Haun F, Nakamura T, Shiu AD, Cho D-H, Tsunemi T, Holland EA (2013). S-Nitrosylation of dynamin-related protein 1 mediates mutant huntingtin-induced mitochondrial fragmentation and neuronal injury in Huntington’s disease. Antioxid Redox Signal.

[23] Yeramian A, Martin L, Serrat N, Arpa L, Soler C, Bertran J (2006). Arginine transport via cationic amino acid transporter 2 plays a critical regulatory role in classical or alternative activation of macrophages. J Immunol.

[24] Lee J, Lee SG, Kim KK, Lim YJ, Choi JA, Cho SN (2019). Characterisation of genes differentially expressed in macrophages by virulent and attenuated *Mycobacterium tuberculosis* through RNA-Seq analysis. Sci Rep.

[25] Giovannoni F, Quintana FJ (2020). The role of astrocytes in CNS inflammation. Trends Immunol.

[26] Colombo E, Farina C (2016). Astrocytes: key regulators of neuroinflammation. Trends Immunol.

[27] Ta TT, Dikmen HO, Schilling S, Chausse B, Lewen A, Hollnagel JO (2019). Priming of microglia with IFN-gamma slows neuronal gamma oscillations in situ. Proc Natl Acad Sci USA.

[28] Kojima H, Nakatsubo N, Kikuchi K, Urano Y, Higuchi T, Tanaka J (1998). Direct evidence of NO production in rat hippocampus and cortex using a new fluorescent indicator: DAF-2 DA. NeuroReport.

[29] Forrester MT, Foster MW, Benhar M, Stamler JS (2009). Detection of protein S-nitrosylation with the biotin-switch technique. Free Radic Biol Med.

[30] Martin M (2011). Cutadapt removes adapter sequences from high-throughput sequencing reads. EMBnetjournal.

[31] Kim D, Langmead B, Salzberg SL (2015). HISAT: a fast spliced aligner with low memory requirements. Nat Methods.

[32] Pertea M, Pertea GM, Antonescu CM, Chang TC, Mendell JT, Salzberg SL (2015). StringTie enables improved reconstruction of a transcriptome from RNA-seq reads. Nat Biotechnol.

[33] Love MI, Huber W, Anders S (2014). Moderated estimation of fold change and dispersion for RNA-seq data with DESeq2. Genome Biol.

[34] Robinson MD, McCarthy DJ, Smyth GK (2010). edgeR: a Bioconductor package for differential expression analysis of digital gene expression data. Bioinformatics.

[35] Lefebvre A, Ma D, Kessenbrock K, Lawson DA, Digman MA (2021). Automated segmentation and tracking of mitochondria in live-cell time-lapse images. Nat Methods.

[36] Stuart EA, King G, Imai K, Ho D (2011). MatchIt: nonparametric preprocessing for parametric causal inference. J Stat Softw.

[37] Harris KL, Kuan WL, Mason SL, Barker RA (2020). Antidopaminergic treatment is associated with reduced chorea and irritability but impaired cognition in Huntington’s disease (Enroll-HD). J Neurol Neurosurg Psychiatry.

[38] Li L, Liu H, Dong P, Li D, Legant WR, Grimm JB (2016). Real-time imaging of Huntingtin aggregates diverting target search and gene transcription. Elife.

[39] Eshraghi M, Karunadharma PP, Blin J, Shahani N, Ricci EP, Michel A (2021). Mutant Huntingtin stalls ribosomes and represses protein synthesis in a cellular model of Huntington disease. Nature Commun.

[40] Sharma M, Rajendrarao S, Shahani N, Ramirez-Jarquin UN, Subramaniam S (2020). Cyclic GMP-AMP synthase promotes the inflammatory and autophagy responses in Huntington disease. Proc Natl Acad Sci USA.

[41] Labadorf A, Hoss AG, Lagomarsino V, Latourelle JC, Hadzi TC, Bregu J (2015). RNA sequence analysis of human Huntington disease brain reveals an extensive increase in inflammatory and developmental gene expression. PLoS ONE.

[42] Agus F, Crespo D, Myers RH, Labadorf A (2019). The caudate nucleus undergoes dramatic and unique transcriptional changes in human prodromal Huntington’s disease brain. BMC Med Genom.

[43] Al-Dalahmah O, Sosunov AA, Shaik A, Ofori K, Liu Y, Vonsattel JP (2020). Single-nucleus RNA-seq identifies Huntington disease astrocyte states. Acta Neuropathol Commun.

[44] Lee H, Fenster RJ, Pineda SS, Gibbs WS, Mohammadi S, Davila-Velderrain J (2020). Cell type-specific transcriptomics reveals that mutant huntingtin leads to mitochondrial RNA release and neuronal innate immune activation. Neuron.

[45] Consortium HDi (2017). Developmental alterations in Huntington’s disease neural cells and pharmacological rescue in cells and mice. Nat Neurosci.

[46] Mahi NA, Najafabadi MF, Pilarczyk M, Kouril M, Medvedovic M (2019). GREIN: an interactive web platform for re-analyzing GEO RNA-seq data. Sci Rep.

[47] Lee J, Ryu H, Ferrante RJ, Morris SM, Ratan RR (2003). Translational control of inducible nitric oxide synthase expression by arginine can explain the arginine paradox. Proc Natl Acad Sci.

[48] Saba J, Couselo FL, Bruno J, Carniglia L, Durand D, Lasaga M (2022). Neuroinflammation in Huntington’s disease: a starring role for astrocyte and microglia. Curr Neuropharmacol.

[49] Paul BD, Sbodio JI, Xu R, Vandiver MS, Cha JY, Snowman AM (2014). Cystathionine gamma-lyase deficiency mediates neurodegeneration in Huntington’s disease. Nature.

[50] Sbodio JI, Snyder SH, Paul BD (2018). Golgi stress response reprograms cysteine metabolism to confer cytoprotection in Huntington’s disease. Proc Natl Acad Sci USA.

[51] Singer E, Walter C, Weber JJ, Krahl AC, Mau-Holzmann UA, Rischert N (2017). Reduced cell size, chromosomal aberration and altered proliferation rates are characteristics and confounding factors in the STHdh cell model of Huntington disease. Sci Rep.

[52] Steffan JS, Kazantsev A, Spasic-Boskovic O, Greenwald M, Zhu YZ, Gohler H (2000). The Huntington’s disease protein interacts with p53 and CREB-binding protein and represses transcription. Proc Natl Acad Sci USA.

[53] Marcora E, Gowan K, Lee JE (2003). Stimulation of NeuroD activity by huntingtin and huntingtin-associated proteins HAP1 and MLK2. Proc Natl Acad Sci USA.

[54] McFarland KN, Huizenga MN, Darnell SB, Sangrey GR, Berezovska O, Cha JH (2014). MeCP2: a novel Huntingtin interactor. Hum Mol Genet.

[55] Zuccato C, Tartari M, Crotti A, Goffredo D, Valenza M, Conti L (2003). Huntingtin interacts with REST/NRSF to modulate the transcription of NRSE-controlled neuronal genes. Nat Genet.

[56] Dunah AW, Jeong H, Griffin A, Kim YM, Standaert DG, Hersch SM (2002). Sp1 and TAFII130 transcriptional activity disrupted in early Huntington’s disease. Science.

[57] Rouillard AD, Gundersen GW, Fernandez NF, Wang Z, Monteiro CD, McDermott MG (2016). The harmonizome: a collection of processed datasets gathered to serve and mine knowledge about genes and proteins. Database.

[58] Visigalli R, Bussolati O, Sala R, Barilli A, Rotoli BM, Parolari A (2004). The stimulation of arginine transport by TNFalpha in human endothelial cells depends on NF-kappaB activation. Biochim Biophys Acta.

[59] Manner CK, Nicholson B, Macleod CL (2003). CAT2 arginine transporter deficiency significantly reduces iNOS-mediated NO production in astrocytes. J Neurochem.

[60] Dioguardi FS (2011). To give or not to give? Lessons from the arginine paradox. J Nutrigenet Nutrigenom.

[61] Jin Y, Liu Y, Nelin LD (2019). Deficiency of cationic amino acid transporter-2 protects mice from hyperoxia-induced lung injury. Am J Physiol Lung Cell Mol Physiol.

[62] Nakamura T, Oh CK, Zhang X, Lipton SA (2021). Protein S-nitrosylation and oxidation contribute to protein misfolding in neurodegeneration. Free Radic Biol Med.

[63] Zaręba-Kozioł M, Szwajda A, Dadlez M, Wysłouch-Cieszyńska A, Lalowski M (2014). Global analysis of S-nitrosylation sites in the wild type (APP) transgenic mouse brain-clues for synaptic pathology. Mol Cell Proteom.

[64] Wijasa TS, Sylvester M, Brocke-Ahmadinejad N, Schwartz S, Santarelli F, Gieselmann V (2020). Quantitative proteomics of synaptosome S-nitrosylation in Alzheimer’s disease. J Neurochem.

[65] Zareba-Koziol M, Bartkowiak-Kaczmarek A, Figiel I, Krzystyniak A, Wojtowicz T, Bijata M (2019). Stress-induced changes in the S-palmitoylation and S-nitrosylation of synaptic proteins. Mol Cell Proteom.

[66] Kartawy M, Khaliulin I, Amal H (2020). Systems biology reveals reprogramming of the S-nitroso-proteome in the cortical and striatal regions of mice during aging process. Sci Rep.

[67] Sarkar S, Korolchuk VI, Renna M, Imarisio S, Fleming A, Williams A (2011). Complex inhibitory effects of nitric oxide on autophagy. Mol Cell.

[68] Tasset I, Sanchez-Lopez F, Aguera E, Fernandez-Bolanos R, Sanchez FM, Cruz-Guerrero A (2012). NGF and nitrosative stress in patients with Huntington’s disease. J Neurol Sci.

[69] Cherubini M, Lopez-Molina L, Gines S (2020). Mitochondrial fission in Huntington’s disease mouse striatum disrupts ER-mitochondria contacts leading to disturbances in Ca(2+) efflux and Reactive Oxygen Species (ROS) homeostasis. Neurobiol Dis.

[70] Chantranupong L, Scaria SM, Saxton RA, Gygi MP, Shen K, Wyant GA (2016). The CASTOR proteins are arginine sensors for the mTORC1 pathway. Cell.

[71] Coman D, Yaplito-Lee J, Boneh A (2008). New indications and controversies in arginine therapy. Clin Nutr.

[72] Deckel AW, Volmer P, Weiner R, Gary KA, Covault J, Sasso D (2000). Dietary arginine alters time of symptom onset in Huntington’s disease transgenic mice. Brain Res.

[73] Minakawa EN, Popiel HA, Tada M, Takahashi T, Yamane H, Saitoh Y (2020). Arginine is a disease modifier for polyQ disease models that stabilizes polyQ protein conformation. Brain.

[74] Singh V, Patel KA, Sharma RK, Patil PR, Joshi AS, Parihar R (2019). Discovery of arginine ethyl ester as polyglutamine aggregation inhibitor: conformational transitioning of huntingtin N-terminus augments aggregation suppression. ACS Chem Neurosci.

[75] Erens C, Van Broeckhoven J, Hoeks C, Schabbauer G, Cheng PN, Chen L (2022). l-Arginine depletion improves spinal cord injury via immunomodulation and nitric oxide reduction. Biomedicines.

[76] Nitschke L, Coffin SL, Xhako E, El-Najjar DB, Orengo JP, Alcala E (2021). Modulation of ATXN1 S776 phosphorylation reveals the importance of allele-specific targeting in SCA1. JCI Insight.

[77] Driessen TM, Lee PJ, Lim J (2018). Molecular pathway analysis towards understanding tissue vulnerability in spinocerebellar ataxia type 1. Elife.

