# Supplementary material for: Elevated SLC7A2 expression is associated with an abnormal neuroinflammatory response and nitrosative stress in Huntington’s disease
Source: J Neuroinflammation. 2024 Feb 28;21:59. doi: 10.1186/s12974-024-03038-2 (PMC10900710; doi:10.1186/s12974-024-03038-2)

Additional File 1

Figure Legends

Fig. S1. Knocking out SLC7A2 by the CRISPR-Cas9 system. A. Scheme showing the three sgRNA sequence targeting exon 2 of the mouse *Slc7a2* gene (pink, guide 1, 2 and 3), the PCR primers for amplification (green, forward and revers primers) and the sequencing primers for Sanger sequencing (orange, sequencing primer). **B.** Confirmation of the large fragment deletion in the SLC7A2KO clones in STHdhQ7 and Q111 cells with Sanger sequencing followed by ICE analysis using the online tool from Synthego. **C.** Intracellular NO levels monitored in real-time using DAF-2DA in STHdhQ7, Q7-SLC7A2KOs, Q111 and Q111-SLC7A2KO in the presence or absence of IFNγ/LPS treatment.

Fig. S2. RNA-seq analysis of transcriptomic changes in STHdhQ7 and Q111 cells in response to IFNγ/LPS treatment. A. PCA plot of all the samples used in the RNA-seq analysis. Q7-C: STHdhQ7 untreated; Q7-C: STHdhQ7 cells with 24-hr IFNγ/LPS treatment; Q111-C: STHdhQ111 untreated; Q111-C: STHdhQ111 cells with 24-hr IFNγ/LPS treatment. **B-C.** KEGG pathway enrichment analysis in STHdhQ7 (**B**) and Q111 (**C**) cells in response to IFNγ/LPS treatment.

Fig. S3. Mitochondrial fragmentation in STHdhQ111 cells in response to IFNγ/LPS treatment. STHdhQ7 and Q111 cells were transfected with mito-turboRFP and treated with IFNγ/LPS for 24 hours in the presence or absence of 1400W hydrochloride. Cells were then fixed in 4% paraformaldehyde with 4% sucrose to preserve mitochondrial morphology. Images were taken with a 60X oil objective (CFI Plan Apochromat Lambda 60X Oil, numerical aperture =1.4) using a laser scanning confocal microscope (Nikon A1R). Scale bar: 5μm.

Fig. S1.

A.

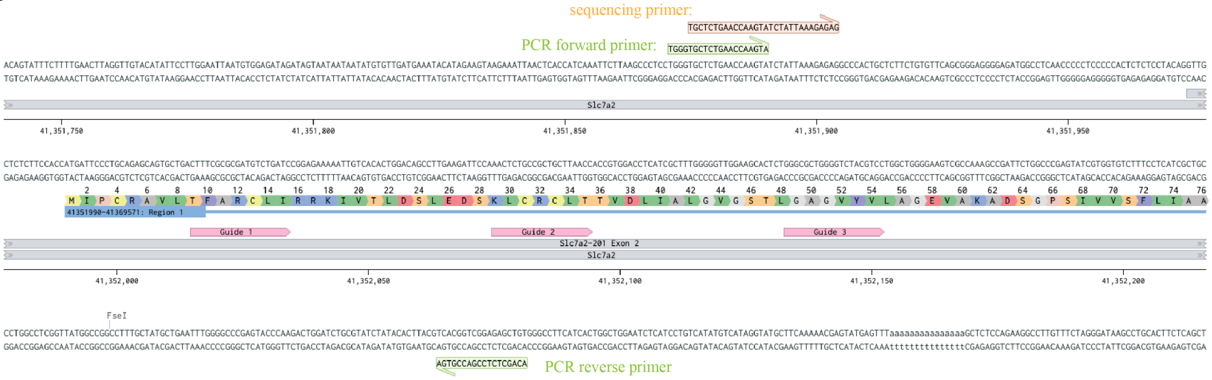

sequencing primer:
TGCTCTGAACCAAGTATCTATTAAAGAGAG
PCR forward primer:
TGGGTGCTCTGAACCAAGTA
Slc7a2
41,351,750
41,351,800
41,351,850
41,351,900
41,351,950
41351990-41369571: Region 1
Guide 1
Guide 2
Guide 3
Slc7a2-201 Exon 2
Slc7a2
41,352,000
41,352,050
41,352,100
41,352,150
41,352,200
FseI
AGTGCCAGCCTCTCGACA
PCR reverse primer

B.

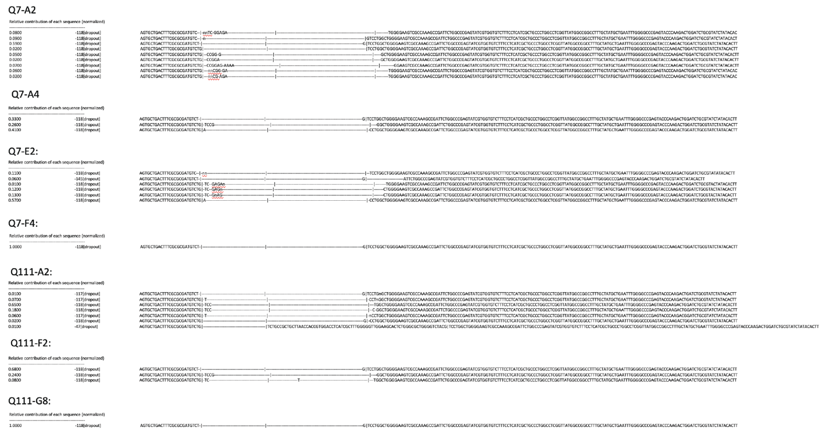

Q7-A2
Q7-A4
Q7-E2:
Q7-F4:
Q111-A2:
Q111-F2:
Q111-G8:

C.

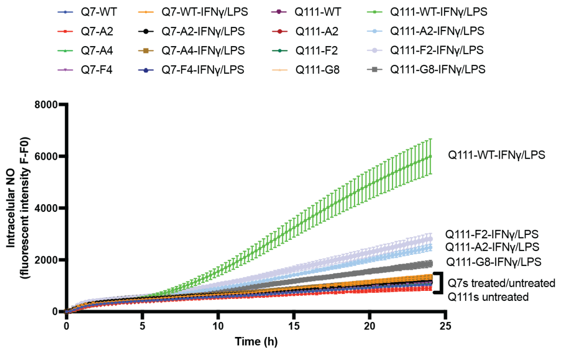

Q7-WT
Q7-WT-IFNγ/LPS
Q111-WT
Q111-WT-IFNγ/LPS
Q7-A2
Q7-A2-IFNγ/LPS
Q111-A2
Q111-A2-IFNγ/LPS
Q7-A4
Q7-A4-IFNγ/LPS
Q111-F2
Q111-F2-IFNγ/LPS
Q7-F4
Q7-F4-IFNγ/LPS
Q111-G8
Q111-G8-IFNγ/LPS
Intracelular NO (fluorescent intensity F-F0)
Time (h)
Q111-WT-IFNγ/LPS
Q111-F2-IFNγ/LPS
Q111-A2-IFNγ/LPS
Q111-G8-IFNγ/LPS
Q7s treated/untreated
Q111s untreated

Fig. S2.

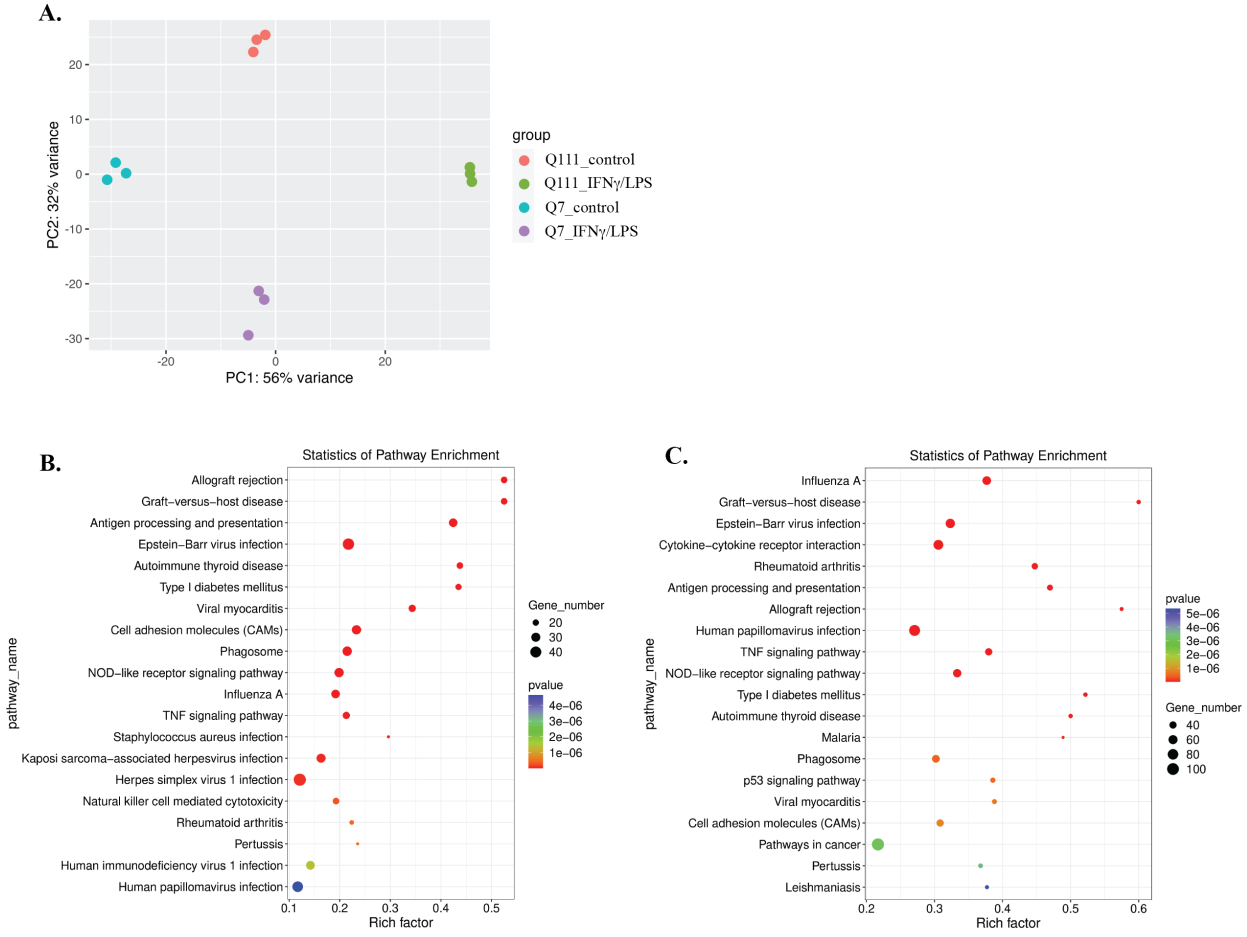

STHdhQ7: IFNγ/control

STHdhQ111: IFNγ/control

Fig. S3.

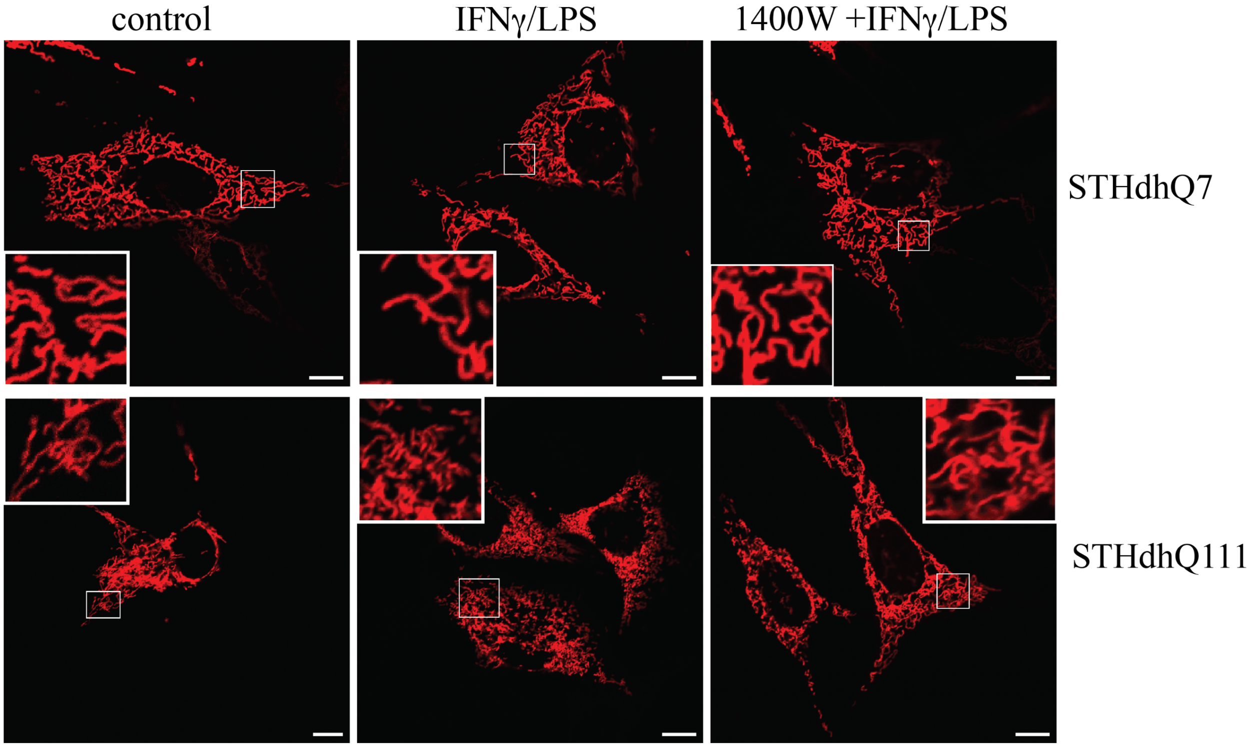

Supplement: Supplementary file 1 — Additional file 1: Figure S1. Knocking out SLC7A2 by the CRISPR–Cas9 system. A Scheme showing the three sgRNA sequence targeting exon 2 of the mouse Slc7a2 gene (pink, guide 1, 2 and 3), the PCR primers for amplification (green, forward and revers primers) and the sequencing primers for Sanger sequencing (orange, sequencing primer). B Confirmation of the large fragment deletion in the SLC7A2KO clones in STHdhQ7 and Q111 cells with Sanger sequencing followed by ICE analysis using the online tool from Synthego. C Intracellular NO levels monitored in real-time using DAF-2DA in STHdhQ7, Q7-SLC7A2KOs, Q111 and Q111-SLC7A2KO in the presence or absence of IFNg/LPS treatment. Figure S2. RNA-seq analysis of transcriptomic changes in STHdhQ7 and Q111 cells in response to IFNg/LPS treatment. A PCA plot of all the samples used in the RNA-seq analysis. Q7-C: STHdhQ7 untreated; Q7-C: STHdhQ7 cells with 24-h IFNg/LPS treatment; Q111-C: STHdhQ111 untreated; Q111-C: STHdhQ111 cells with 24-h IFNg/LPS treatment. B, C KEGG pathway enrichment analysis in STHdhQ7 (B) and Q111 (C) cells in response to IFNg/LPS treatment. Figure S3. Mitochondrial fragmentation in STHdhQ111 cells in response to IFNg/LPS treatment. STHdhQ7 and Q111 cells were transfected with mito-turboRFP and treated with IFNg/LPS for 24 h in the presence or absence of 1400W hydrochloride. Cells were then fixed in 4% paraformaldehyde with 4% sucrose to preserve mitochondrial morphology. Images were taken with a 60× oil objective (CFI Plan Apochromat Lambda 60× Oil, numerical aperture = 1.4) using a laser scanning confocal microscope (Nikon A1R). Scale bar: 5 μm. [file 12974_2024_3038_MOESM1_ESM.pdf]
